# Hierarchical development of dominance through the winner-loser effect and socio-spatial structure

**DOI:** 10.1371/journal.pone.0243877

**Published:** 2022-02-02

**Authors:** Erik van Haeringen, Charlotte Hemelrijk

**Affiliations:** Groningen Institute for Evolutionary Life Sciences, University of Groningen, Groningen, The Netherlands; Universidade de São paulo, BRAZIL

## Abstract

In many groups of animals the dominance hierarchy is linear. What mechanisms underlie this linearity of the dominance hierarchy is under debate. Linearity is often attributed to cognitively sophisticated processes, such as transitive inference and eavesdropping. An alternative explanation is that it develops via the winner-loser effect. This effect implies that after a fight has been decided the winner is more likely to win again, and the loser is more likely to lose again. Although it has been shown that dominance hierarchies may develop via the winner-loser effect, the degree of linearity of such hierarchies is unknown. The aim of the present study is to investigate whether a similar degree of linearity, like in real animals, may emerge as a consequence of the winner-loser effect and the socio-spatial structure of group members. For this purpose, we use the model DomWorld, in which agents group and compete and the outcome of conflicts is self-reinforcing. Here dominance hierarchies are shown to emerge. We analyse the dominance hierarchy, behavioural dynamics and network triad motifs in the model using analytical methods from a previous study on dominance in real hens. We show that when one parameter, representing the intensity of aggression, was set high in the model DomWorld, it reproduced many patterns of hierarchical development typical of groups of hens, such as its high linearity. When omitting from the model the winner-loser effect or spatial location of individuals, this resemblance decreased markedly. We conclude that the combination of the spatial structure and the winner-loser effect provide a plausible alternative for hierarchical linearity to processes that are cognitively more sophisticated. Further research should determine whether the winner-loser effect and spatial structure of group members also explains the characteristics of hierarchical development in other species with a different dominance style than hens.

## Introduction

Dominance hierarchies are a near universal pattern of social order in group-living animals [1]. High rank is considered adaptive for access to resources and protection from predators [2–6]. The hierarchy is often (near) linear in small groups of up to 10 individuals in a wide range of species, including mammals, fish, birds, crustacean and insects [1, 7]. Yet, what proximate mechanisms cause linearity is under longstanding scientific debate.

Two mechanisms have been proposed for hierarchy formation, the prior attributes hypothesis and self-organisation hypothesis. The prior attributes hypothesis proposes that attributes individuals possess prior to hierarchy formation, such as body size or correlated traits, directly determine their rank position in a linear hierarchy [7]. This theory is supported by empirical data on pair-wise dominance interactions [8], but has been rejected in some theoretical studies because for prior attributes to produce (near) linear hierarchies difficult mathematical conditions would be required, especially in larger groups [9–11]. Namely, attributes of fighting of many individuals in the group are expected to lay around the mean. Therefore, many individuals have an intermediate chance to win from those with similar fighting power. This similarity in winning tendency is likely to lead to cyclical dominance relationships. For example, after the pair-wise contests among three individuals of similar fighting ability it could be that individual A has won from B, B from C and C from A. Because the differences in attributes among individuals decrease with group size, Chase and Lindquist [11] argue it is unlikely that differences in prior attributes alone directly produce (near) linear hierarchies.

The second hypothesis proposes that hierarchy formation is the result of self-reinforcing effects in experience of individuals that arise during the hierarchy formation [12]. The self-reinforcing effect implies that, the winner of a dominance interaction is more likely to win again, whereas the loser is more likely to lose again, the so-called winner-loser effect [13–15]. The winner-loser effect operates in a wide variety of species, including insects, crustacean, spiders, fish, birds and mammals [16]. Most evidence of the influence of winner-loser effects on hierarchy formation comes from contests in experimental studies of isolated pairs, rather than in a group [7, 11, 17]. Here the differences in individual attributes are minimized to isolate the winner-loser effect. A recent exception is a study that provided the first evidence in a wild and uncontrolled population of primates (baboons) for the role of the winner-loser effect in the dynamics of the hierarchy using novel statistical methods [18]. Some other examples of the winner-loser effect in groups include a study in crabs [19] and in quails [20].

Another study that examined hierarchy formation in a group is that by Lindquist and Chase [21]. The authors tracked the hierarchy of small groups of hens by analysing patterns in dominance behaviour and the development of the dominance network over time. They showed that the hierarchy became highly linear and stable and it developed fast. Hens attacked each other in series (bursts) and the network states (configurations of dominance relationships in the group) that occurred most often were those that either contained an individual that dominated all others, or that comprised only triads that were transitive (shown in a triad motif analysis of the network). The authors mathematically represented three models of hierarchy development based on the winner-loser effect. Namely, the Bonabeau model [22], the Dugatkin model [15] and the Hemelrijk model, called DomWorld [23]. However, Lindquist and Chase ignored the spatial representation in the model DomWorld [21]. Instead, they investigated whether their mathematical abstractions of the three models reproduce some aspects of the hierarchy formation in hens. The authors concluded that neither the winner-loser effect nor prior attributes directly account for the formation of linear hierarchies. As to the winner-loser effect, because none of the model abstractions based on it could mimic the linear hierarchy observed in hens. As to prior attributes, because differences in attributes in earlier theoretical and experimental work could not explain the linearity of the hierarchy in groups, Lindquist and Chase argue that the patterns of agonistic behaviour and the instability of intransitive states of the dominance network indicate instead that hens are aware of the group hierarchy and actively strive to make it linear through processes that are cognitively sophisticated. The authors suggest that to understand the linearity of some dominance hierarchies, cognitive processes like transitive inference [24], eavesdropping [25] and individual recognition [26] are necessary.

The detailed description of Lindquist and Chase of the formation of dominance hierarchies in hens offers an opportunity to examine for the first time whether the DomWorld model generates hierarchical patterns similar to those in hens, despite the model’s cognitively simple rules (agents are not striving for linearity of the dominance hierarchy). We here investigate the importance of the spatial representation of interactions for the formation of a highly linear and stable hierarchy, because the spatial component of the model in combination with the winner-loser effect has formerly been shown to contribute to a wide variety of complex patterns of social interaction resembling those in primates, including many aspects of egalitarian and despotic dominance styles of various species of macaques [27, 28].

Where the winner-loser effect alone could not explain the development of the highly linear hierarchy of hens, we hypothesize that combining it with a spatial component will lead to a more stable and linear hierarchy. We expect this to arise from the behaviour where those that lose and thus flee more often consequently spend more time at the outside of the group, whereas the dominant individuals cluster in the centre. This results in individuals interacting more frequently with others of similar rank, lowering the chance of reversals in the hierarchy [23].

Therefore, the aim of the present study is to investigate how the winner-loser effect and the socio-spatial structure affect the development of the dominance hierarchy in the model DomWorld. The use of a computational model allows us to investigate how the different processes, separately and combined, contribute to the observed hierarchical traits. For this, we compare the hierarchy formation in the model DomWorld in the presence and absence of the winner-loser effect and of the spatial component while using methods similar to those in hens by Lindquist and Chase [21]. Next, we investigate in DomWorld whether the winner-loser effect combined with a socio-spatial component results in dominance hierarchies that are highly linear and stable as observed in hens.

## Methods

### The model

The computer model DomWorld [23] is an individual-based model in which agents move in infinite space. The agents have a tendency to group when other agents are far away and engage in dominance interactions when other agents are within their ‘personal space’. DomWorld is event-driven and does not have a representation of time. Dominance interactions between agents can be either won or lost. The outcome of a fight is self-reinforcing, such that the winner becomes more likely to win subsequent fights and the loser more likely to lose these. Throughout this article we will use the terms ‘*win’* and ‘*lose’* for the outcome of dominance interactions and ‘*initiation’* for starting a dominance interaction, also referred to as a fight. Fights in DomWorld represent a range of antagonistic behaviours including non-physical interactions such as approach-retreat interactions. Different intensities of a fight (such as approach-retreat or a physical fight) are represented in the impact a fight has. The impact is higher when the parameter that controls the intensity of aggression (*StepDom*) is greater and when the outcome is less expected.

A dominance interaction is mediated by dominance values (DOM) that represent each agent’s fighting power. The chance *W*_*i*_ of agent *i* to win a fight against agent *j* is determined by comparing its ratio of the *DOM* values to a number drawn from a random distribution.


$$W_{i}=\left\{\begin{array}{l} 1\;\frac{{DOM}_{i}}{{DOM}_{i}+{DOM}_{j}}\\ 0\;else \end{array}>RND(0,1\right)$$ (1)

Afterwards the *DOM* values are updated depending on the outcome of the fight. The value of the winner (*DOM*_*i*_) increases with one minus its relative dominance ratio. The loser decreases its score by the same amount. The change in DOM value is multiplied by a scaling parameter *StepDom* that symbolises the intensity of aggression.


DOMi=DOMi+(Wi−DOMiDOMi+DOMj)*StepDomDOMj=DOMj−(Wi−DOMjDOMi+DOMj)*StepDom (2)

In the model, DomWorld, agents have been using two strategies of attack [23], obligate and risk-sensitive. In the present paper we will only discuss the obligate style of attack, as risk-sensitivity in general did not influence the patterns of hierarchy formation and also was not used in the work by Lindquist and Chase [21]. When agents are meeting an individual in their personal space and are set to always attack it, this is referred to as an obligate strategy of attack [27]. If an agent attacks only if it assumes it will win from its opponent, this has been called risk-sensitive attack. Here, an agent will first mentally simulate the fight with its opponent (Eq 1). Only if the agent wins its mental fight it will initiate an actual fight. Note that for the risk-sensitive style of attack the assumption is required that the agent knows or estimates both the dominance score of its opponent and itself. This is not the case for the obligate style of attack for which the agent only requires an internal record or feeling of success that is updated by the outcome of actual fights. A more extensive description of DomWorld can be found in Hemelrijk [23].

To gain understanding of the effects of spatial structure and the winner-loser effect we studied three versions of the DomWorld model. The first is the full model. The second is a version without the spatial component of DomWorld. Where in the full model agents move about and interact with others nearby, in the version without the spatial component agents select an interaction partner at random. The third is a version without the winner loser effect. Where in the full model the DOM score (representing fighting power) of an agent is updated after a fight, in the version without the winner-loser effect the DOM scores are fixed. The values of the DOM scores are set to the final scores of the simulations with the full model.

### The measures

In analysing the development of the dominance hierarchy in DomWorld our measures and naming conventions are similar to those by Lindquist and Chase for groups of hens [21], see below.

#### Hierarchy

We determine the dominance hierarchy in the group using the Average Dominance Index (ADI) which is the average of an individual’s proportions of wins when interacting with each of its interactions partners in the group [29]. Note that the ADI was found to perform (almost) the same as the computationally more complex David score [29], that similarly to the ADI determines sum of the ratios of winning and losing per dyad. It weighs the result by the power of the opponent, which equals the sum of its winning ratios like in the ADI. We calculated the ADI cumulatively after each interaction. The agents are ranked based on the ADI, where the agent with the highest value (i.e. highest average proportion of winning from its group members) is the most dominant. We measure the stability of the dominance hierarchy by counting the number of rank-changes. Since Lindquist and Chase found in hens that the majority of the rank-changes occurred during the early stages of formation and remained mostly stable afterwards, we also measured rank changes during the first 60 interactions and during the entire run.

Note that the hierarchies are established based on the outcome of fights like in empirical data, not on the DOM scores of the agents in the DomWorld model. Therefore, the order of the hierarchy that is measured in the model version of DomWorld without the winner-loser effect can change over time even though the fighting power (DOM) of the agents is fixed in this version.

Further, we characterise the differentiation of the hierarchy in DomWorld by the steepness measure from de Vries et al. [30]. Here, steepness is determined by fitting a linear regression to the ADI of the agents plotted against the rank of the agents using the ordinary least square method, where the slope of the line is the steepness of the hierarchy.

Lindquist and Chase do not report the steepness of the hierarchy in hens. To compare the hierarchy in DomWorld to that of hens we estimated the average steepness measure of de Vries for the groups of hens using the average dominance values reported in Fig 10 of Lindquist and Chase [21]. For this we used imaging software to sample 11 points per individual from this figure. Ten of these were taken from interaction 50 to 500 (one per 50 interactions). The first point was sampled after 10 interactions instead of zero, when there is no hierarchy.

#### Patterns in dominance behaviour

In their observations of the hierarchy formation in hens, Lindquist and Chase identified two striking patterns in dominance behaviour which they suggest may be found in other social species as well [21]. The first is the burst, which is defined as a series of consecutive attacks (at least two) in the same direction in a single pair of individuals. Important to note is that this definition does not involve a time interval. Attacks that are part of a burst can theoretically be separated widely in time, as long as there are no attacks involving other pairs in between. In hens the burst was abundant, sometimes involving a long series of attacks. Here the lack of counter attacks suggests clear differences in dominance among the hens. To quantitatively examine these patterns in DomWorld we recorded the number of interactions that was part of a burst and determined the maximum length of a single burst per run.

The second pattern is called a pair-flip. A pair-flip occurs in a dyad when the direction of a subsequent attack is opposite to that of the previous one. In hens, pair-flips were clustered in the first phase of the trial and rarely coincided with rank-changes. A pair-flip was often immediately followed by a counter pair-flip. The immediate retaliation and lack of pair-flips later in the trials suggest the hierarchy is stable and dominance relations are clear. We examined similarly in DomWorld by the total number of pair-flips per run as well as during the first 60 interactions and the percentage of rank-changes that coincided with a pair-flip.

In nature, access to a valuable resource such as food or safety often depends on the hierarchical position of an individual. When dominant individuals are free to go anywhere while subordinates are continuously chased away, individuals become assorted by rank. This assortment promotes the linearity and stability of the hierarchy because there are fewer encounters between individuals further away in rank [23]. This spatial centrality of dominant individuals, we measured per activation (from activation 0 to 60) in DomWorld by correlating the dominance rank of each individual with its distance to the centre of the group.

#### Structure of the dominance network

**Fig 1:**
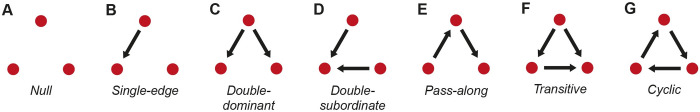
Motifs of directed relations in a triad. Motifs A to E are partial triads, motifs F and G are complete triads. Individuals in motif F can be ranked. Therefore, motif F is transitive. Individuals in motif G cannot be ranked and therefore, it is intransitive (cyclic).

We describe the development of the dominance network using a network analysis of triad motifs, where the triads are all the possible sets of three individuals that can be formed in the network [31, 32]. Each motif comprises three individuals (nodes) and their three dyadic relationships (one relationship is called a link or an edge). Each triad in the network is labelled according to the presence and direction of dominance relationships among its members. When considering only directed relations, there are seven triad motifs (Fig 1). If each individual has a directional relationship with two members of the triad, the triad can either be transitive (Fig 1F) or intransitive (Fig 1G). Transitive implies that A dominates both B and C, B dominates C, and C is subordinate to both. Cyclic or intransitive implies that while A dominates B, B dominates C and C dominates A.

**Fig 2:**
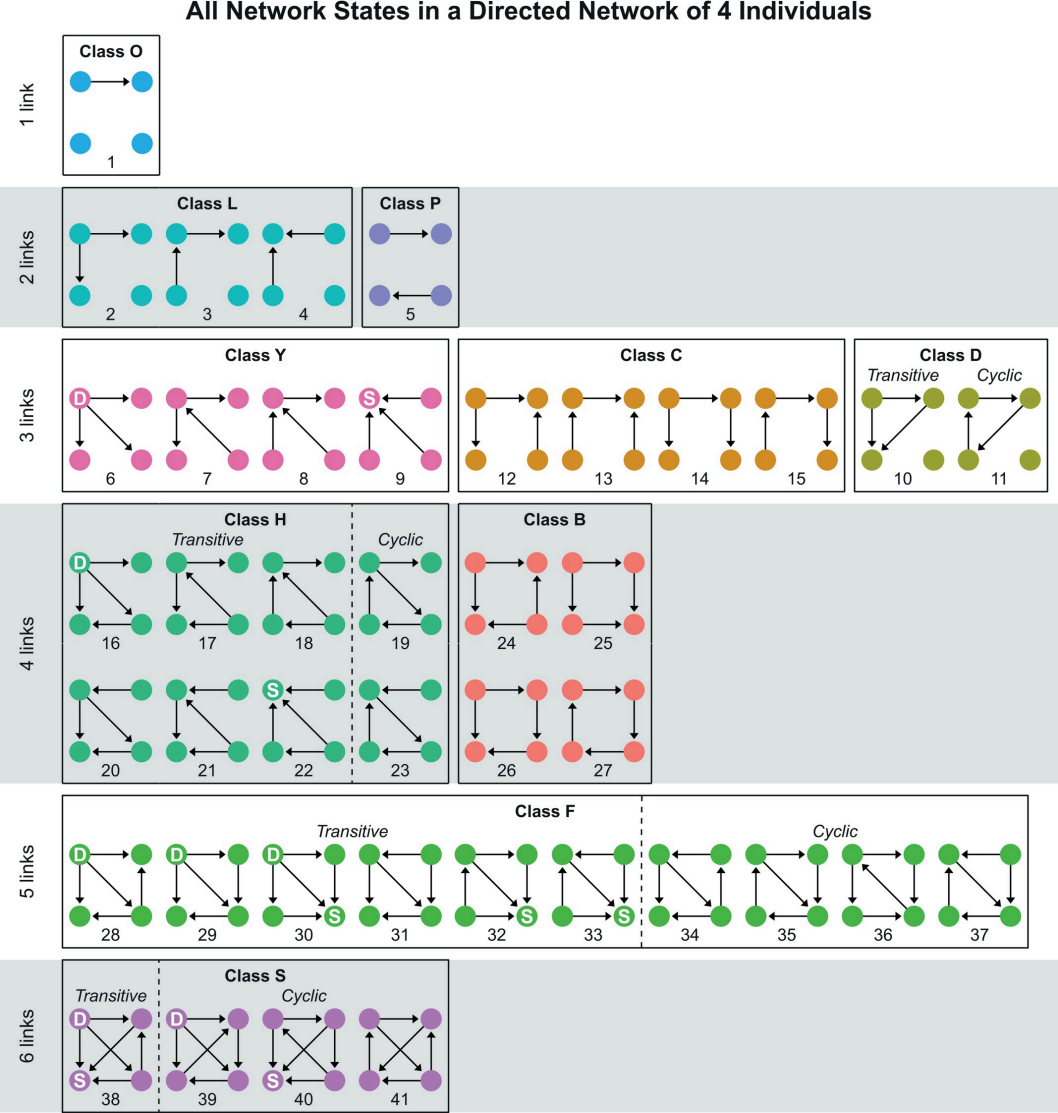
All states of a directed network of four individuals. The states are categorised by number of links (edges), by class and transitivity, following [21]. Note that the colours of the classes correspond to the colours in Fig 7. A node marked with a ‘D’ is an individual that Dominates All Others (DAO), and marked with an ‘S’ is an individual Submissive to All Others (SAO).

The configuration of all relationships in a network is called a (network) state, and the entire set of all possible configurations in a network is called the state space. In a directed network with four individuals there are four triads and the state space includes 41 different states that are shown in Fig 2, plus one arbitrary state with no relations among individuals that is not included. States are categorised in groups with the same number of dyadic relations in the network, the so-called link-group (1-link, 2-links etc.). In a link-group states are categorised in classes that are indicated by a letter (class B, class F etc.). States in the same class share a network structure such that a pair-flip can change the network state to another one in the same class, but not to a network state in a different class (or link-group). If there is an individual that is dominant over all others (indicated as DAO) in the group, its node is marked with a ‘D’. The node of an individual that is submissive to all others (SAO) is marked with an ‘S’. The development of the network is traced over time by recording its state after each interaction.

Linearity of a social network is measured as the transitivity of triads. We measured the degree of transitivity in two ways. The first, used by Lindquist and Chase in hens, is the proportion of states that are completely transitive without any cyclic triad (called transitive states) out of all states with at least one complete triad. Second, we also calculated the transitivity measure T_tri_, as described by Shizuka and McDonald [32]. This is the proportion of complete triads (not states) that are transitive, normalised by the proportion transitive triads that are expected on average in a random network. Because a state can also be partially transitive (i.e., include both transitive and cyclic triads), in theory this proportional measure of transitivity has a higher resolution than its binary definition of a state being either completely transitive or cyclic. Since results for the binary and proportional definition of triad transitivity were similar for the settings in the present paper, we only show the proportion of transitive triads, T_tri_.

The occurrence of each network state is examined using two measures: the Class Occurrence Frequency (COF), which is the proportion of simulations in which a state occurred at least once, and the Class Stability Frequency (CSF), which is the number of interactions that occurred while the dominance network was in a particular state, divided by the total number of interactions that occurred in all the states with the same number of links. For each state both measures (COF and CSF) are shown in a histogram categorised according to the link-group and the class with the number of recorded interactions to indicate the degree of accuracy (Fig 7).

### Setup

**Fig 3:**
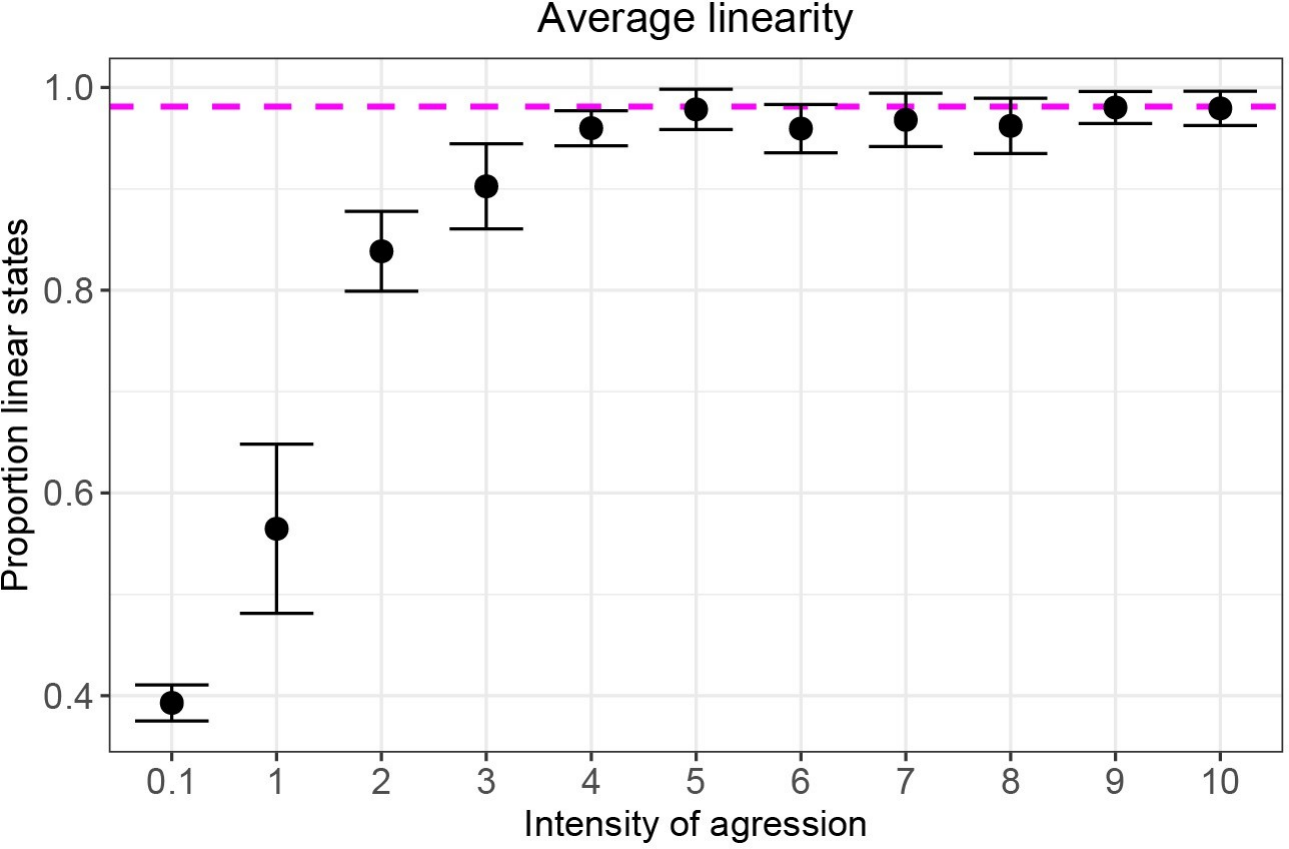
Average (and SE) of the hierarchical linearity measured as the proportion of transitive states for different levels of intensity of aggression. The dashed line indicates the proportion of transitive states estimated for the groups of hens in Lindquist and Chase [21].

The parameter setting of DomWorld from Hemelrijk in 1999 [33] functioned as a starting point for the present study. This included the initial density and DOM values of the agents, distances and angles related to perception and navigation and the size of the personal space. Next we tuned the model DomWorld to match several aspects of competition among real hens observed by Lindquist and Chase [21]. Namely, we used the same number of groups (14 groups), the same group size (four females) and the same average number of interactions (518 interactions) as used in the study of hens [8]. To obtain in the model the same average linearity of the hierarchy as reported in hens, we increased the value of a single parameter, *StepDom*, to represent a higher intensity of aggression, thus, increasing the average linearity of the hierarchy in the model (Fig 3).

We estimated linearity in the empirical data of hens from Fig 12 of the study of Lindquist and Chase from the frequencies [21]. Our estimation within the states with the same number of links concerns the proportion of network states that is fully linear. In 95% of the interactions in DomWorld the network contained the maximum of six links. Because in states with six links, state 38 is the only fully transitive state, we chose its proportion as our target value to tune StepDom. State 38 occurred in hens in 98% of the interactions with six links (see dashed line Fig 3). For the present paper we chose the lowest value of StepDom that matched this target, which is a value of StepDom of nine.

Another difference with the parameter setting in Hemelrijk [33] is that in the present paper we examine the dominance interactions from the start of the run to study the process of hierarchy development, while Hemelrijk skipped the transient period and focussed on dominance interactions when the hierarchy was already formed. A full list of parameter settings is included in S1 Appendix.

### Analysis

#### Data collection

For each condition data were collected over 14 repetitions with the model DomWorld while running it for 518 steps. Note that in the case of omitting the winner-loser effect from the model these 14 runs differed from each other since the fixed DOM scores of the agents were set to the final DOM scores of different runs of full version. The main analysis of the three versions of the model included 42 runs. An additional 140 runs were performed with the full DomWorld model for tuning the intensity of aggression as described in the previous section. In our data-analysis of DomWorld we used scripts written in Python (version 3.6.8) for the various measures, that we made available in the DHDAT package [34].

#### Statistical analysis and figures

The statistical analysis and creation of figures were performed with R (version 3.5.1) and RStudio (version 1.2) in combination with packages Ggplot2, Multcomp, CAR and Dplyr. All quantitative measures reported in the present paper are given as the average and the standard error over 14 runs with the exception of the music notation plots. They show the development of the hierarchy over a single run.

We statistically tested the effects of removing the spatial component and of removing the winner-loser effect from the DomWorld model on nine quantitative measures, using generalized linear models (GLM). For each measure we first created a full statistical model where the measure was the response variable and the fixed variables included the version of DomWorld that was used (complete, without the winner-loser effect and without space) and the identifier of the run (Table 1). For analysing hierarchy steepness, the maximum length of bursts and the proportion of pair-flips and rank-changes during early hierarchy formation (items 3, 4, 6 and 8 of Table 1), we used a Gaussian distribution in the GLM, while for all other parameters we used a binomial distribution. For the models with a Gaussian distribution, we visually checked the assumptions regarding the distribution of the residuals and the homogeneity of variance with the help of scatterplots of the model as provided by R. To reduce the positive skewness of hierarchy steepness (item 3 in Table 1) we applied a log transformation. We tested for collinearity between the fixed variables by calculating the variance inflation factor using the CAR package in RStudio and determined there was no significant collinearity as all results were below three, the recommended threshold by Zuur et al. [35]. We also checked for outliers using Cook’s distance with a threshold of 4/N. When we repeated the analysis after removing the outliers, this yielded the same conclusions in all cases. Therefore, we show the results of the models including the outliers.

**Table 1:** Overview of the GLM models with their response variables, fixed variables in the full model, fixed variables after model reduction (minimal adequate model) and the distribution that was used.

	Response variable	Fixed variables	Minimal adequate model	Distribution
1)	Rank-changes (% of interactions)	Model version + run number	Model version	Binomial
2)	Rank-changes correlated with pair-flips	Model version + run number	Model version	Binomial
3)	Rank-changes during first 60 interactions	Model version + run number	-	Gaussian
4)	Hierarchy differentiation (steepness)	Model version + run number	Model version + run number	Gaussian
5)	Pair-flips (% of interactions)	Model version + run number	Model version + run number	Binomial
6)	Pair-flips during first 60 interactions	Model version + run number	-	Gaussian
7)	Bursts (% of interactions)	Model version + run number	Model version	Binomial
8)	Maximum burst length	Model version + run number	Model version	Gaussian
9)	Average linearity (proportion fully transitive states)	Model version + run number	Model version + run number	Binomial

When starting from the nine full statistical models we determined the minimal adequate model for each by performing an analysis of variance (ANOVA) and continuing to drop the predictor variable with the highest p-value until all predictor variables left in the model had a p-value below the significance level of 0.05. In order to determine which model versions significantly differed from one another, we applied a Tukey test post hoc to the minimal adequate model of each measure.

Removing the spatial component or the winner-loser effect from the model had a significant effect on all measures shown in Table 1, except for the proportion of rank-changes and pair-flips early in the formation (items 3 and 6 of Table 1) for which no minimal adequate model was found.

The values shown in the results section in Table 2 are averages and standard error (SE) measured over 14 runs. The last column shows the test results (GLM) of the effect of the model version on the respective response variable. The results from the post-hoc Tukey tests are shown as letters (a, b, c) in superscript. If a letter is not shared between two values this indicates a significant difference between these values. For instance, the proportion of rank-changes (item 1 in Table 2) is significantly lower in the full model than in the model without winner-loser effect. If a letter is shared, there is no significant difference between these groups, e.g. considering the proportion of rank-changes, between the full model and that without the spatial component.

**Table 2:** Quantitative measures of dominance behaviour and network for the three versions of the model, namely the full model, the model without the spatial component, and without the winner-loser effect.

		Full model		Without spatial component		Without winner-loser effect		
* *	Measure	*Value*	*SE*	*Value*	*SE*	*Value*	*SE*	P-value
1)	Rank-changes (% of interactions)	1.7%^a^	±0.2	2.2%^ab^	±0.2	2.4%^b^	±0.2	0.01
2)	Rank-changes correlated with pair-flips	16.4%^a^	±3.7	21.1%^a^	±3.6	42.3%^b^	±5.0	< 0.001
3)	Rank-changes during first 60 interactions	54.6%	±5.2	61.2%	±4.5	58.8%	±7.9	0.74
4)	Hierarchy differentiation (steepness)	-0.29^b^	±0.00	-0.25^a^	±0.00	-0.30^b^	±0.00	< 0.001
5)	Pair-flips (% of interactions)	3.9%^a^	±0.2	7.8%^b^	±0.3	8.5%^b^	±0.3	< 0.001
6)	Pair-flips during first 60 interactions	9.1%	±3.3	11.5%	±1.6	14.2%	±5.0	0.29
7)	Bursts (% of interactions)	52.3%^c^	±0.6	28.3%^a^	±0.5	48.3%^b^	±0.6	< 0.001
8)	Maximum burst length	11.2^b^	±0.7	4.1^a^	±0.1	10.3^b^	±0.8	< 0.001
9)	Average linearity (proportion fully transitive states)	0.98^b^	±0.00	0.99^c^	±0.00	0.96^a^	±0.00	< 0.001

The hierarchy is established using the average dominance index (ADI). The nine measures correspond to the response variables of the statistical models in Table 1. The results of the Tukey test are shown as superscript letters (a, b, c). When a letter is not shared between two model versions this indicates a significant difference between them.

## Results

### 1. Behavioural dynamics

#### 1.1. Rank development

In hens, Lindquist and Chase found that the hierarchy was highly differentiated and stable [21]. Rank-changes were few and happened mostly during the first stage of hierarchy formation. For instance, a top-ranking individual often emerged early and subsequently maintained its position. Most promotions in rank came from an agent attacking another that was lower in rank, which increased its dominance score until it surpassed that of an individual ranking above itself. Rank-changes were seldom directly preceded by a reversal in the direction of attack in that pair (pair-flip).

**Fig 4:**
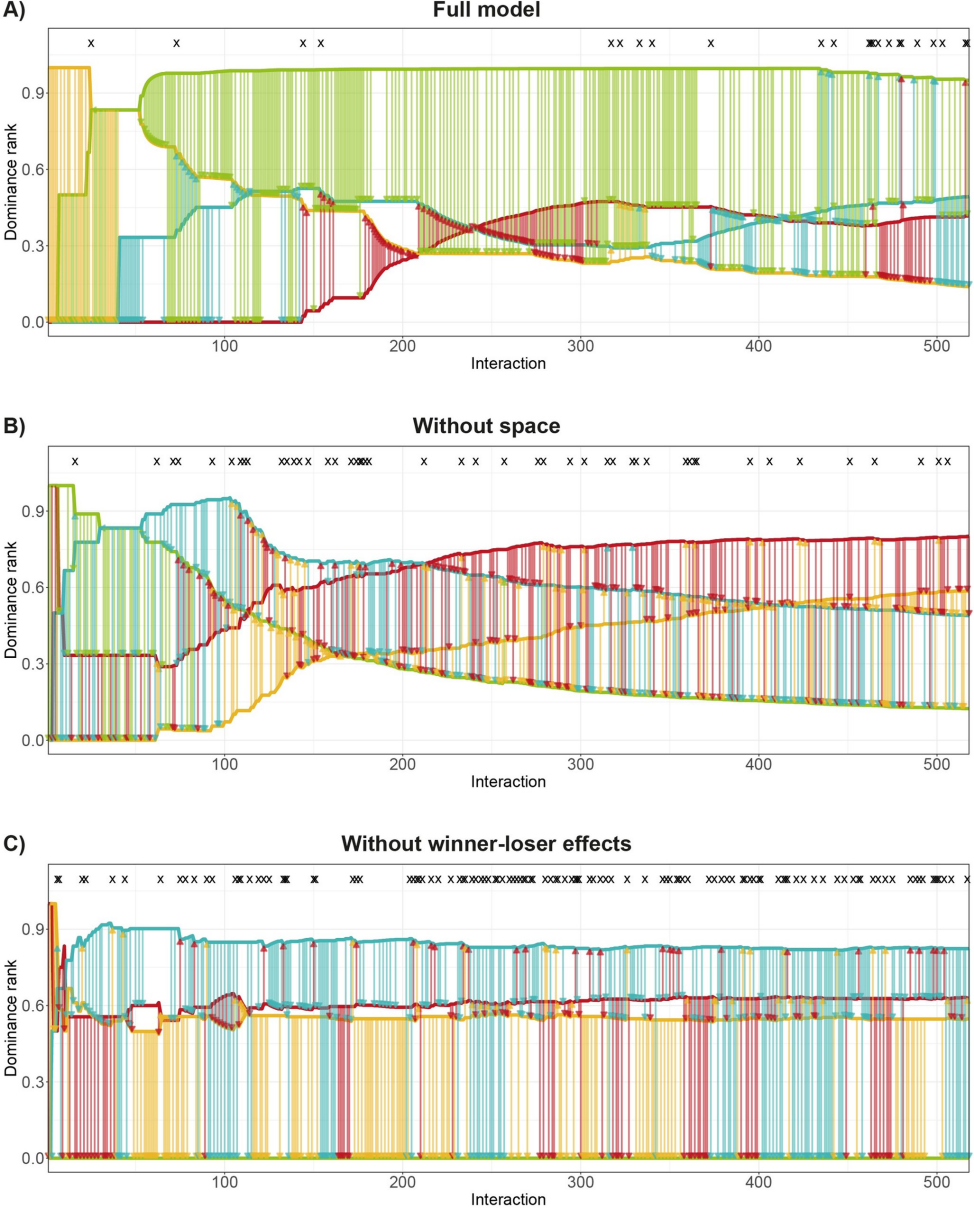
Music notation graph of rank development over interaction count for A) the full DomWorld model, and the DomWorld model B) without the spatial component and C) without the winner-loser effect. The lines represent the rank of each individual based on the average dominance index (ADI). The vertical arrows represent separate fights and point from the winner to the loser, in the colour of the winner. Pair-flips are marked with an ‘X’ at the top of the graph. Rank-changes occurred at the points where two or more lines cross each other.

Rank development in DomWorld (full model) is characterized by its stability (item 1 in Table 2) and the strength of the differentiation of the hierarchy (4 in Table 2). The top-ranking individual often emerged early in the run (in 10 of the 14 runs, see S2 Appendix), maintaining its rank throughout. Rank-changes that involved individuals at lower rank positions are distributed more uniformly over time (Fig 4).

Without a spatial representation or without the winner-loser effect the model leads to more changes of rank (1 in Table 2). Additionally, without a spatial representation when meeting others randomly, rank-changes more often involve all rank positions over the entire length of the run (S2 Appendix), and result in a hierarchy that is less steep than in the full version of the model and the model without the winner-loser effect (4 in Table 2).

In all model versions more than half of the ascensions in rank occurred during the first 60 interactions (3 in Table 2). In the leadup to a rank-change, the increase in the dominance value of the individual that will ascent in rank comes about by a combination of attacking lower ranking individuals and attacking the individual ranking immediately above itself with which it will swap rank, but not often by attacking others that are much higher in rank (Fig 4, also see S2 Appendix). Only one in six rank-changes in the full model is directly preceded by a pair-flip (2 in Table 2). When removing the winner-loser effect from the model, rank-changes more often correlate with a pair-flip than in the full model (2 in Table 2).

#### 1.2. Pair-flips

An interaction is classified as a pair-flip when a loser from an interaction wins from the same opponent in the subsequent fight. Hence a lower frequency of pair-flips indicates a more stable hierarchy. In hens, Lindquist and Chase reported that pair-flips were scarce, and half of them occurred during the first 60 interactions [21]. Pair-flips were often quickly followed by another pair-flip indicating immediate retaliation of aggression.

In the full DomWorld model pair-flips occurred about half as often as in the model without space or without the winner-loser effect (5 in Table 2). In contrast to the pattern in hens, pair-flips in DomWorld (all versions) were not concentrated during early hierarchy formation (6 in Table 2) and were usually not directly followed by a counter pair-flip (Fig 4).

#### 1.3. Bursts

Attacks (pecks) by hens were often repetitive in the study by Lindquist and Chase [21], involving one individual attacking the same opponent several times in a series. The duration of these ‘bursts’ followed a power-law with a maximum length of around 120 repetitive pecks. Pecks during bursts were directed down the hierarchy by all individuals except the lowest ranking individual. This is obvious because it does not have any individual lower in rank to attack.

**Fig 5:**
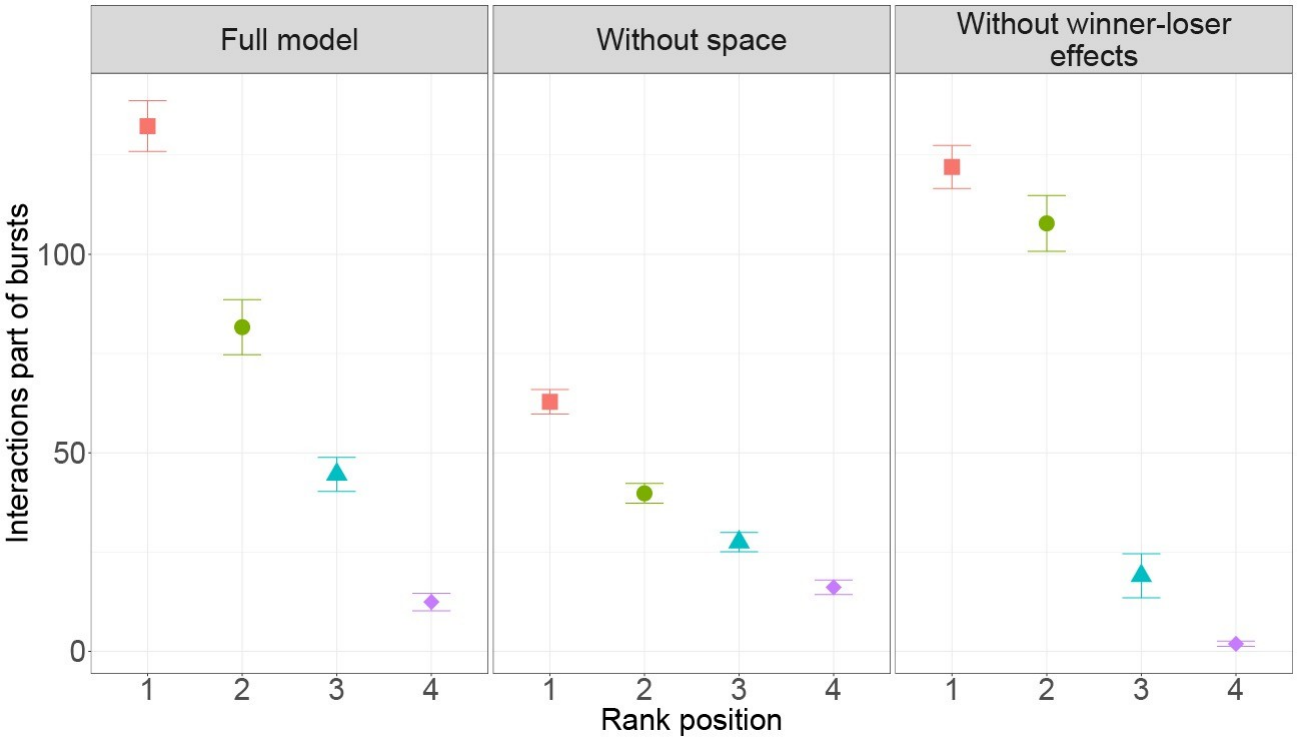
Average (and standard error, SE) number of interactions part of bursts per run. This is shown for each model version per rank position (rank 1 is most dominant).

About 50% of the interactions are part of bursts in the full model and in DomWorld without the winner-loser effect (7 in Table 2), with a maximum length of about 10–11 consecutive interactions (8 in Table 2). Without space in DomWorld, the percentage of interactions involved in bursts is halved and the maximum length of a burst reduces to approximately four interactions. In all model versions the average number of interactions in a burst was higher the greater the dominance of the attacker (Fig 5).

#### 1.4. Spatial distribution

**Fig 6:**
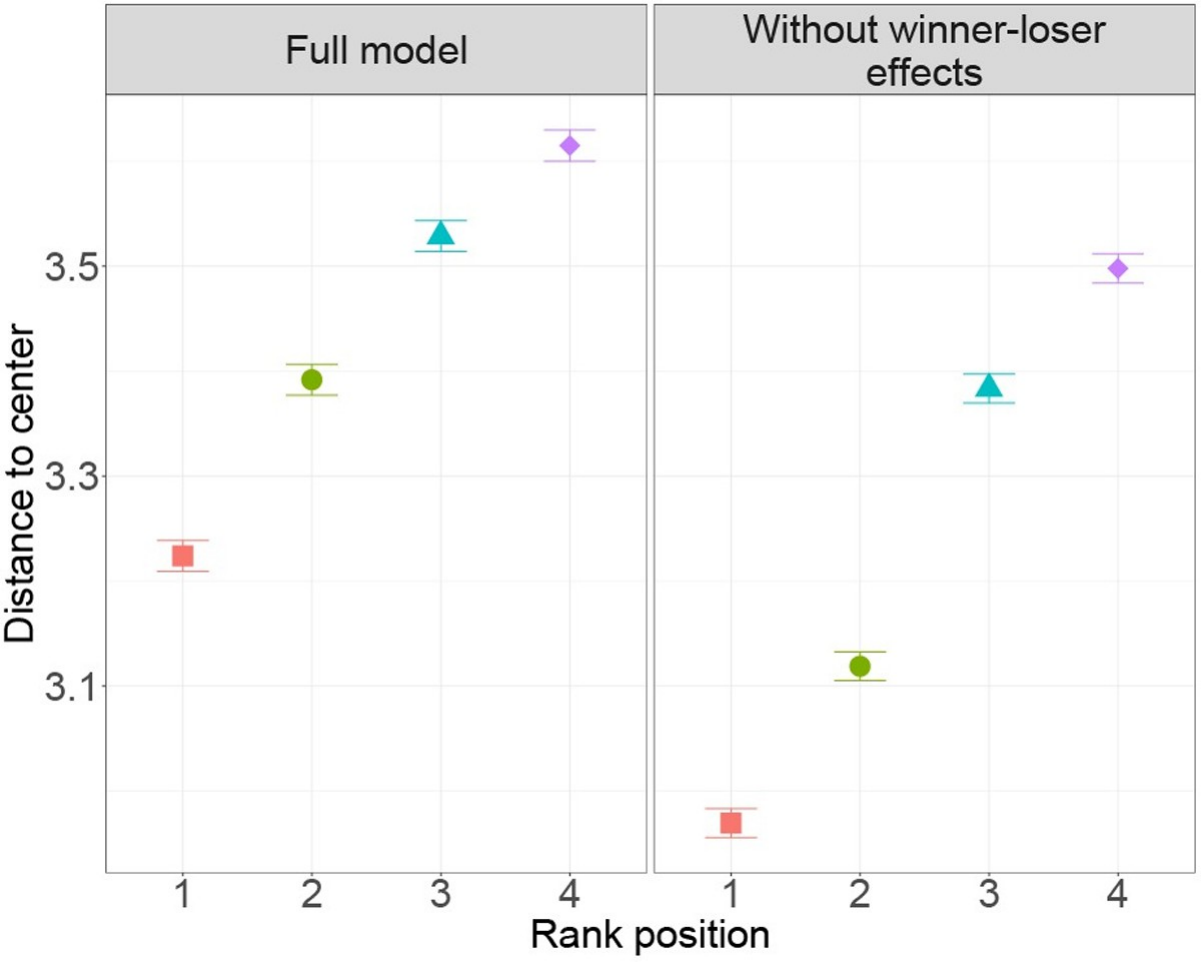
Average (and standard error, SE) distance to the centre of the group. This is shown per rank position (rank 1 is most dominant) for the full model and the model without the winner-loser effect.

Even though a group size of four individuals is small, a spatial structure still emerged in DomWorld in which the dominant individual is more often in the centre of the group, while the lower ranking individuals are on average further away from it (Fig 6).

### 2. Network analysis of triad motifs

To investigate the development of the dominance network as a whole, all its triadic combinations of individuals are assigned a triad motif. The collection of all triad motifs forms the state space of the network, see the section *Patterns of network structure* in *Methods*. The network of groups of hens in the study of Lindquist and Chase [21] developed rapidly via different paths until they reached a complete network with six links. Here those states occurred more often that either comprised of one or more transitive triads, no cyclic triads or one individual that is dominant over all others (DAO).

**Fig 7:**
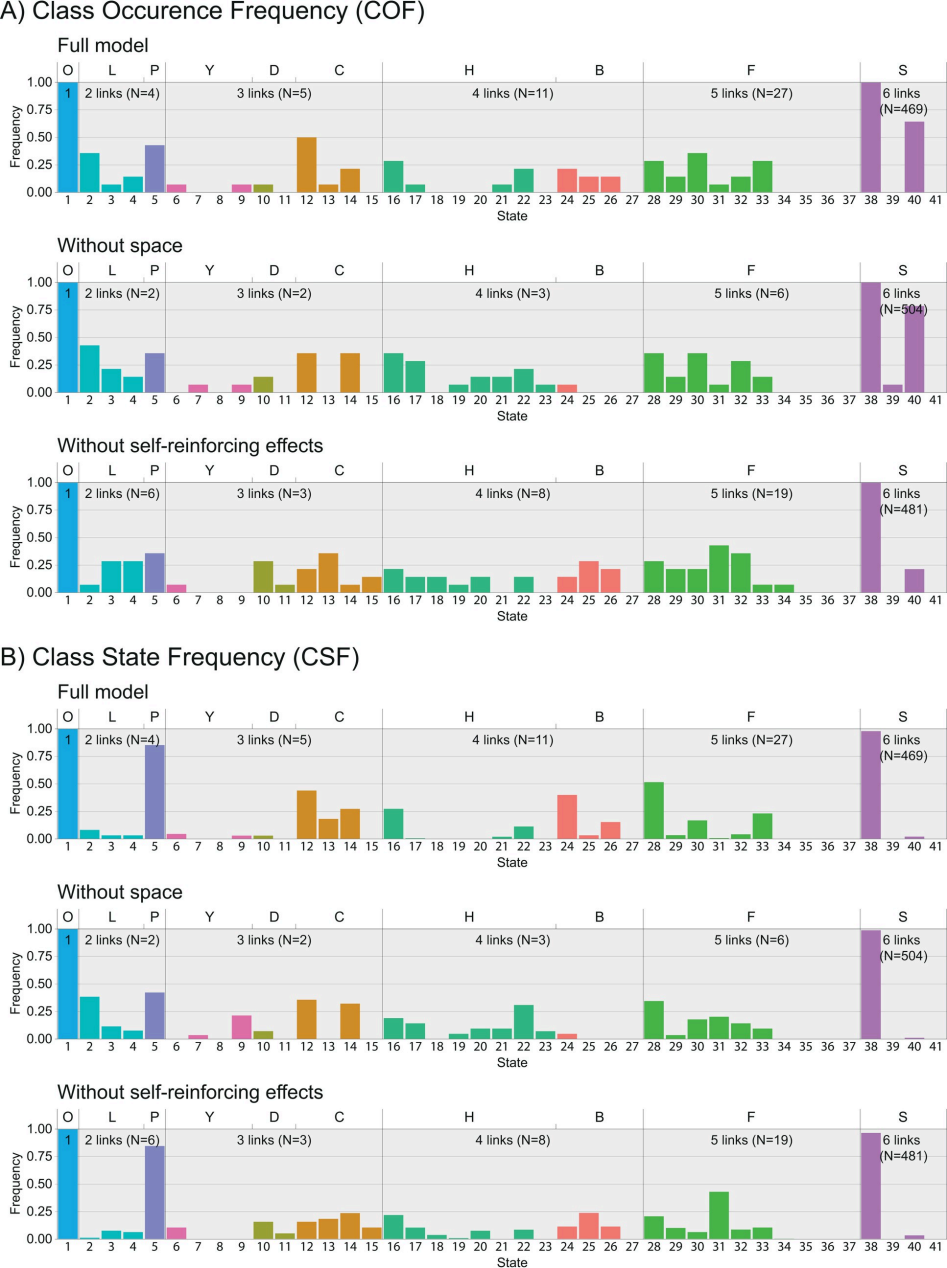
Histogram of A) in how many groups did a state occur at least once (Class occurrence frequency, COF) and B) how many times did a state occur over all runs relative to the total occurrence of states with the same number of links (Class state frequency, CSF). The state index (bottom), class letter (top) and colours correspond to those in Fig 2. Note that neither Class occurrence frequency, COF nor Class state frequency, CSF reflects the absolute number of the occurrence of a state. For each link-class the average number of interactions per run is shown as ‘N’ to give an indication of accuracy.

Similar to hens, the complete network of six links was reached fast in DomWorld. Less than one tenth of the total interactions resulted in a state with less than six links in each of the model versions (see N for each link-class in Fig 7). Also, the developmental path through the network states until a completely connected state was reached varied among runs. Together this makes it difficult to draw conclusions from the initial development of the hierarchy when the network is incomplete (states with less than six links). Therefore, in the present paper we will focus on states with six links.

Of the four states with six links (Fig 2), state 38 is the only one that is fully transitive, with four transitive triads. State 39 and 40 comprise one cyclic triad and three transitive triads, and state 41 has two cyclic triads and two transitive triads. State 38 and 39 are the only states that have an individual that dominates all others (DAO), whereas state 38 and 40 contain an individual who is submissive to all others.

The frequencies of triad motifs in the original model and its two derived versions are similar (Fig 7B). Almost all (96–99%) interactions result in a network with state 38. The abundance of state 38 implies a high proportion transitive states, indicating the linearity of the hierarchy. The difference in frequencies of state 38 across the three model versions directly contributes to significant but small difference in their proportion of transitive states (9 in Table 2). Of the other states with six links, state 40 is the most common in all model versions (1–4%) and is together with state 38 the only state with six links that contains an individual that is submissive to all others. States 39 and 41 occur in is less than 1% of all interactions.

## Discussion

**Fig 8:**
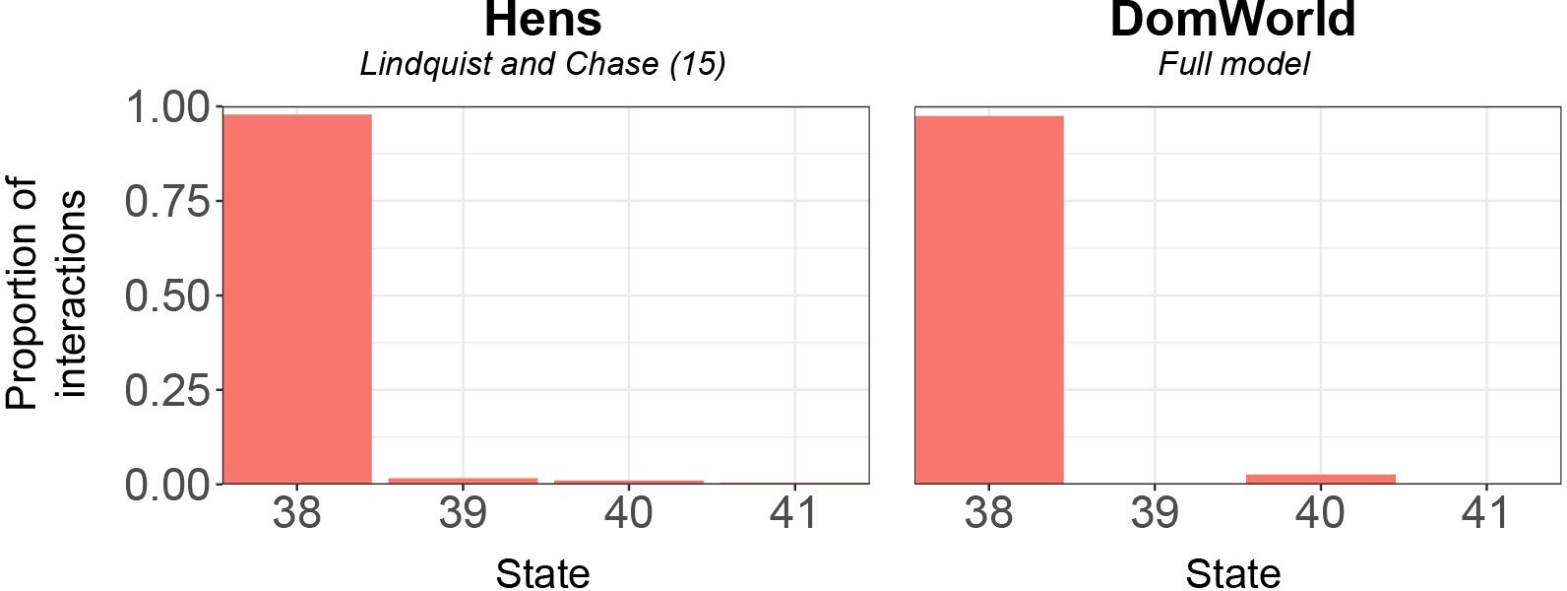
Comparison of the frequency of complete states (six links) in the dominance network between the model DomWorld and hens as reported by Lindquist and Chase [21].

As to the similarity in hierarchy development between groups in DomWorld and real hens [21], we showed that groups in DomWorld developed a highly linear and stable hierarchy with characteristics similar to those of hens (Table 3). After the value of the parameter *intensity of aggression* was increased compared to former settings that were relevant to macaques, the hierarchical linearity in the model was similar to that in hens (11 in Table 3). Also the frequency of rank-changes, pair-flips and bursts in the model resembled those in hens (2, 5 & 7 in Table 3). The hierarchy in DomWorld developed rapidly whereby most changes in rank occurred early in the development of the hierarchy and soon most network states were fully connected with six links. The frequency distribution of the complete network states (with six links) in DomWorld resembled that of hens (Fig 8). A difference is that in DomWorld intransitivity is mostly the result of state 40, whereas in hens intransitivity comes from all states that contain one or more cyclic triads, thus also from states 39 and 41.

**Table 3:** Comparison between patterns of hierarchical development in the full model DomWorld and in groups of hens [21].

* *	Measure	DomWorld	Hens
		*Full model*	*Lindquist and Chase [21]*
1)	Hierarchy differentiation	Steep (R = -0.29)	Steep (R ≈ -0.29)
2)	Rank-changes per run	Few (8.7/run)	Few
3)	Rank position is determined rapidly	Yes (55% rank-changes in first 60 interactions)	Yes
4)	Top-ranking individual often emerges early and consolidates its position while late rank-changes occur in lower ranks	Yes (10/14 runs)	Yes
5)	Bursts (proportion of fights)	0.52	0.49
6)	Maximum duration of bursts	17 interactions	~120 interactions
7)	Pair-flips (proportion of fights)	0.04	0.02
8)	Percentage pair-flips during early development (< 60 interactions)	9%	51%
9)	Pair-flip is often quickly followed by a counter pair-flip	No	Yes
10)	Rank-changes correlate with pair-flips	Few (16.4%)	No
11)	Average linearity (proportion transitive states)	0.98	~0.98

A few behavioural patterns in hens were absent in DomWorld. Most notably the long series of repeated attacks within a pair (6 in Table 3) and the immediate retaliation of aggression described for hens were absent (9 in Table 3). Further, pair-flips in DomWorld were more evenly distributed over the length of the run, whereas in hens they clustered during early hierarchy development (8 in Table 3). An explanation for these differences might lie in two methodological problems of matching the behaviour of individuals in our computational model to empirical data.

First, the nature of the dominance interactions that are simulated in DomWorld only coarsely match the level of detail of the behaviour observed in hens. Lindquist and Chase recorded at the level of individual acts of aggression in hens that predominantly consisted of pecks. In DomWorld dominance interactions represent complete fights, with an initiator that starts the fight, and a winner and a loser, where the loser flees after the fight. Thus, the lower level of detail of fights in DomWorld could explain the absence of very long series of individual attacks (bursts) and the immediate retaliation to aggression of a lower ranking individual that were found in hens.

Second, the spatial environment of individuals in the model differs significantly from that of the groups of hens. The hens were confined in a cage [21, 36], whereas in DomWorld agents moved through an unlimited space [33]. Thus in DomWorld the loser of a fight, often subordinate to the attacker, may flee without restriction, whereas in hens an individual is limited by the border of the cage. Since this hinders the subordinate hen to flee its attacker, it is more likely to suffer a longer series of attacks. This contributes to the higher frequency and duration of bursts in hens. The lack of escape options after a pair-flip in hens may also have promoted counter aggression, resulting in an immediate counter pair-flip, a pattern that was observed in hens but we did not find in DomWorld. To investigate these hypotheses, future studies should examine hierarchy formation in hens without the confines of a cage.

The lack of clustering in time of pair-flips in DomWorld as opposed to in hens may be due to the limits we set to dominance scores in the model. If dominance scores in DomWorld differ more between individuals this decreases the chance of a pair-flip. During hierarchy formation in DomWorld dominance scores were often restricted by these limits. Because all individuals started with the same dominance score which typically diverges over time, dominance score are less often limited during the initial part of the formation. Therefore, widening these limits in the model would mainly decrease the number of pair-flips later in the run and thus increase clustering of pair-flips during the first stage of hierarchy formation, as was reported for hens. Furthermore, asymmetrical clipping of the highest dominance scores might explain the relatively high frequency of state 40 in DomWorld compared to hens. This is because state 38 transforms to state 40 via a pair-flip that involves the highest-ranking individual, whereas for state 38 to transform to state 39 it requires a pair-flip involving the individual with the lowest rank, and to transform to state 41 involves a pair-flip between both the lowest and highest rank (see Fig 2). Thus if the chosen limits more often restrict the dominance score of the lowest ranking individual than that of the highest ranking individual this may promote the number of pair-flips involving the lowest ranking individual.

Based on their review of the literature and a comparison among three well-known winner-loser models and observations of real hens, Lindquist and Chase [21] concluded that the winner-loser effect is not sufficient to explain development of linear hierarchies in groups of hens. The authors suggest that hens instead are aware of the hierarchy and their own position in it and are striving to keep the hierarchy linear. For this, the authors propose processes that are cognitively sophisticated, such as transitive inference and eavesdropping. These processes are absent in DomWorld.

Transitive inference, with which individuals fill in transitive relationships for unobserved relationships, has indeed been found in a wide variety of species, including cognitively simpler species such as hens and recently even insects [37]. Where transitive inference was long thought to be the hallmark of human reasoning, the ability of simpler species to solve transitive-inference tasks begs the question whether the mechanism underlying transitive-inference-like behaviour is truly cognitively demanding [38]. Yet while cognitively simpler explanations have been proposed based on reinforcement history [39, 40], experimental evidence is lacking [41–44].

However, it is unclear whether the task commonly used to measure transitive inference is directly relevant to the social context of real animals such as dominance relations in a group [38, 45]. The vast majority of evidence for transitive inference in animals has been collected with the so-called N-term series task, wherein animals are first trained and then tested using transitive series of arbitrary stimuli such as colours, odours or shapes. A study that illustrates this question, by Takahashi and colleagues [46], finds that three species (tree shrews, rats and mice), which in other studies were shown to solve the N-term series task [24, 47], were not able to solve two inference tasks in social context, while a fourth species (capuchin monkeys) could.

In the present study we show that the winner-loser effect in combination with a socio-spatial component successfully reproduces many of the characteristics of hierarchy development in hens without the need for cognitively sophisticated processes, such as transitive inference. Thereby it forms a plausible alternative to assuming the need of transitive inference in dominance processes. Removing the winner-loser effect from DomWorld, thus representing fixed individual capacities of winning, reduces the resemblance of the model to interactions patterns in hens compared to the full model. Furthermore, by experimenting with the presence of the spatial configuration of group members in the model we show that spatial interaction is essential for the formation of a highly linear and stable hierarchy.

On the other hand, even though the model DomWorld shows patterns of hierarchy development resembling those in real hens, it cannot prove the existence of underlying processes in real animals. Also, in the present paper we focused on the winner loser effect by starting all individuals with the same fighting capacity, but in reality the winner-loser effect and the effects of prior attributes are not mutually exclusive and together affect the hierarchy. Although challenging, future research should determine the role of socio-spatial structure in hierarchy formation in real animals, including wild populations.

In recent years a broader call has been echoed to investigate the development of social networks over time, arguing that for testing hypotheses relevant for selection, dynamics, development and evolution of social networks, it is necessary to include temporal dynamics and spatial constraints [48–51]. Along these lines further research may focus on collecting time-series of data of development of the hierarchy in other species in order to determine whether the combination of the winner-loser effect and the socio-spatial structure can generally explain the formation of linear dominance hierarchies, also in species with different dominance styles than hens.

## Supporting information

S1 Appendix Parameter settings DomWorld. (PDF)

S2 Appendix Full set of music notation graphs. (PDF)
