# Supplementary material for: Hierarchical development of dominance through the winner-loser effect and socio-spatial structure
Source: PLoS One. 2022 Feb 2;17(2):e0243877. doi: 10.1371/journal.pone.0243877 (PMC8809560; doi:10.1371/journal.pone.0243877)
Supplement: S1 Appendix — (PDF) [file pone.0243877.s001.pdf]

APPENDIX 1. FULL LIST OF PARAMETER SETTINGS USED IN DOMWORLD

Note: Male specific parameters are omitted as only runs with females were performed.

Parameter	Setting
Periods	*60*
First data period	*0*
Period duration factor	*20*
Number of runs	*14*
Number of females	*4*
Initial density	*1.7*
Initial dominance score	*8*
Minimum DOM score	*0.0001*
Maximum DOM score	*48*
Intensity of aggression (StepDom)	*Varied between 0.1 and 10*
Risk sensitivity (Alpha)	*0*
Field of view	*120*
Personal space distance	*2*
Near view distance	*24*
Max view distance	*50*
Flee distance	*2*
Withdraw distance	*0*
Chase distance	*1*
Move distance	*1*
Wiggle turn angle	*0*
Wiggle turn error	*10*
Search turn angle	*90*
Search turn error	*10*
Won turn angle	*45*
Won turn error	*0*
Flee turn angle	*180*
Flee turn error	*10*
