# Supplementary material for: Hierarchical development of dominance through the winner-loser effect and socio-spatial structure
Source: PLoS One. 2022 Feb 2;17(2):e0243877. doi: 10.1371/journal.pone.0243877 (PMC8809560; doi:10.1371/journal.pone.0243877)
Supplement: S2 Appendix — (PDF) [file pone.0243877.s002.pdf]

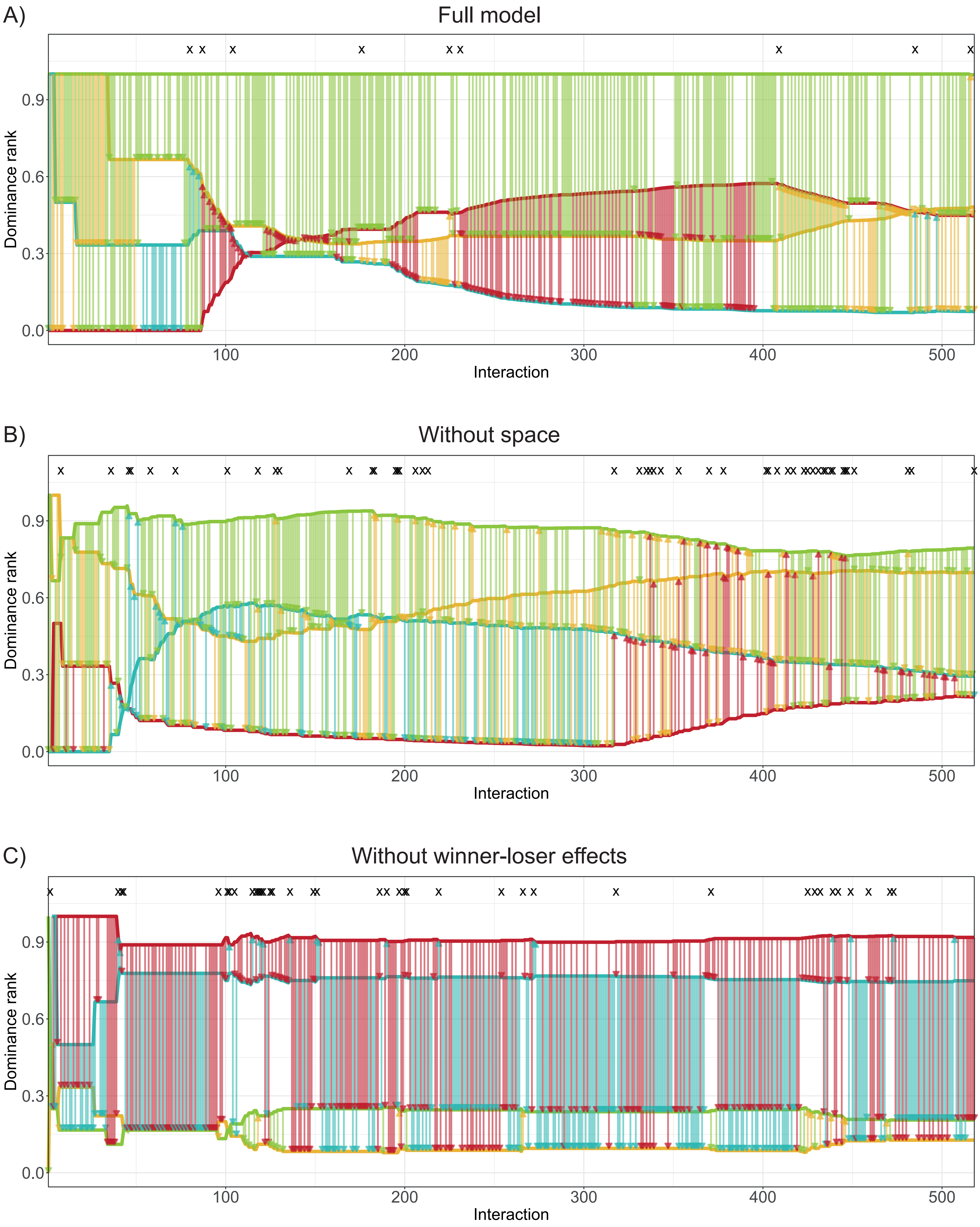

Appendix 2, Run 1. Music notation graph of rank development over interaction count for A) the full DomWorld model, B) without the spatial component and C) without the winner-loser effect. The horizontal lines represent the rank of each individual based on the average dominance index. The vertical arrows represent fights pointing from the winner to the loser, in the colour of the winner. Pair-flips are marked with an ‘X’ at the top of the graph. Rank changes are shown as crossing horizontal lines.

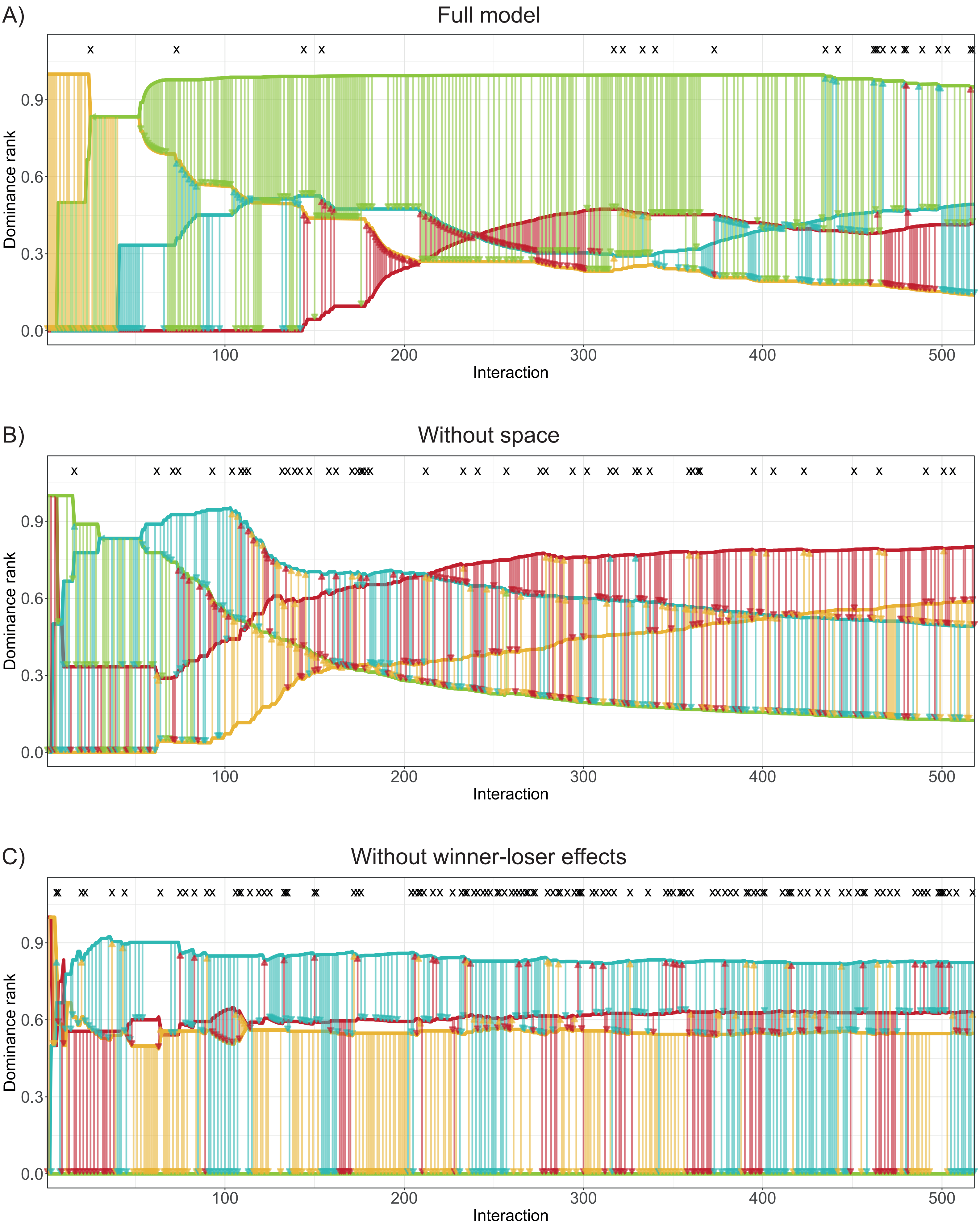

Appendix 2, Run 2. Music notation graph of rank development over interaction count for A) the full DomWorld model, B) without the spatial component and C) without the winner-loser effect. The horizontal lines represent the rank of each individual based on the average dominance index. The vertical arrows represent fights pointing from the winner to the loser, in the colour of the winner. Pair-flips are marked with an ‘X’ at the top of the graph. Rank changes are shown as crossing horizontal lines.

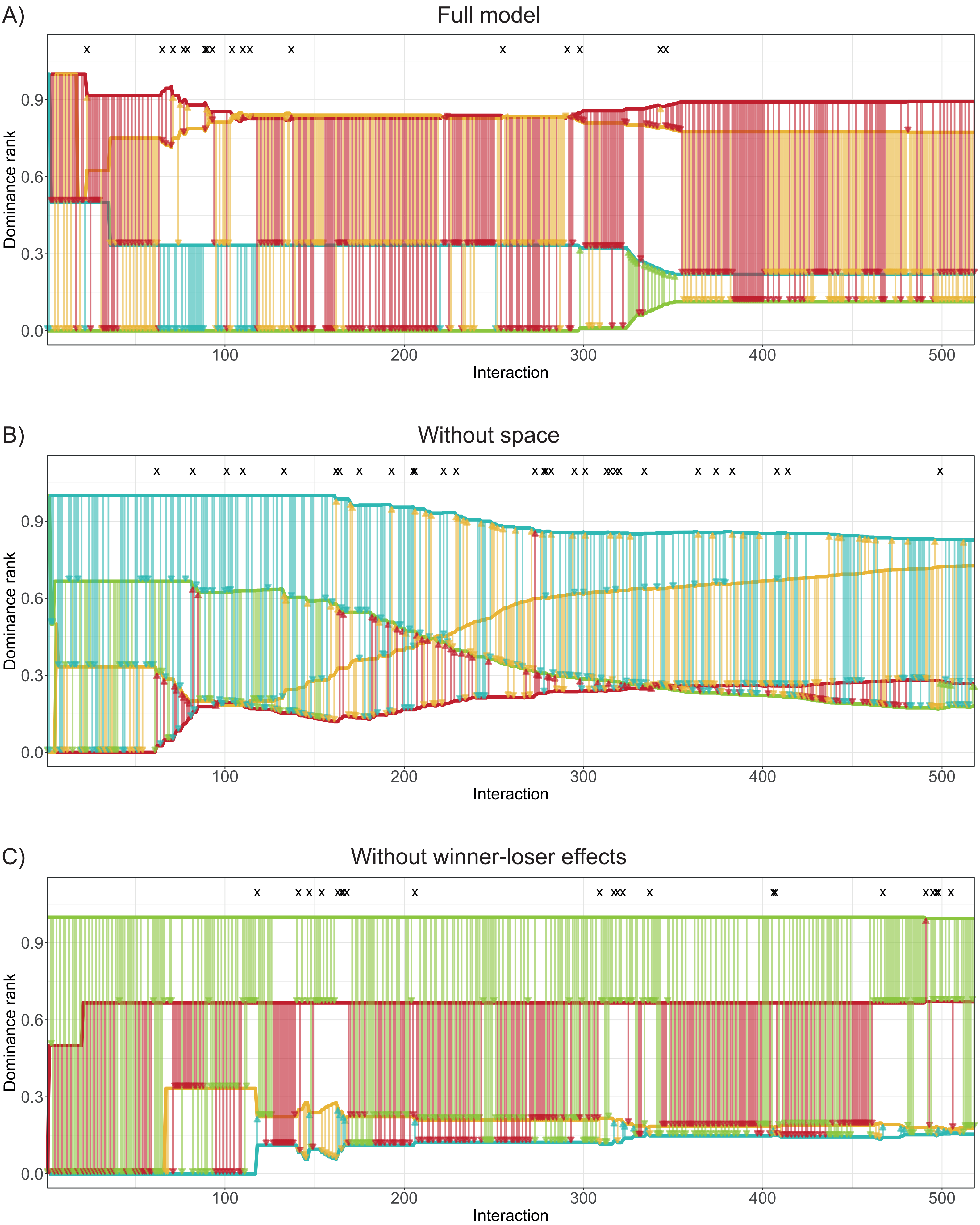

Appendix 2, Run 3. Music notation graph of rank development over interaction count for A) the full DomWorld model, B) without the spatial component and C) without the winner-loser effect. The horizontal lines represent the rank of each individual based on the average dominance index. The vertical arrows represent fights pointing from the winner to the loser, in the colour of the winner. Pair-flips are marked with an ‘X’ at the top of the graph. Rank changes are shown as crossing horizontal lines.

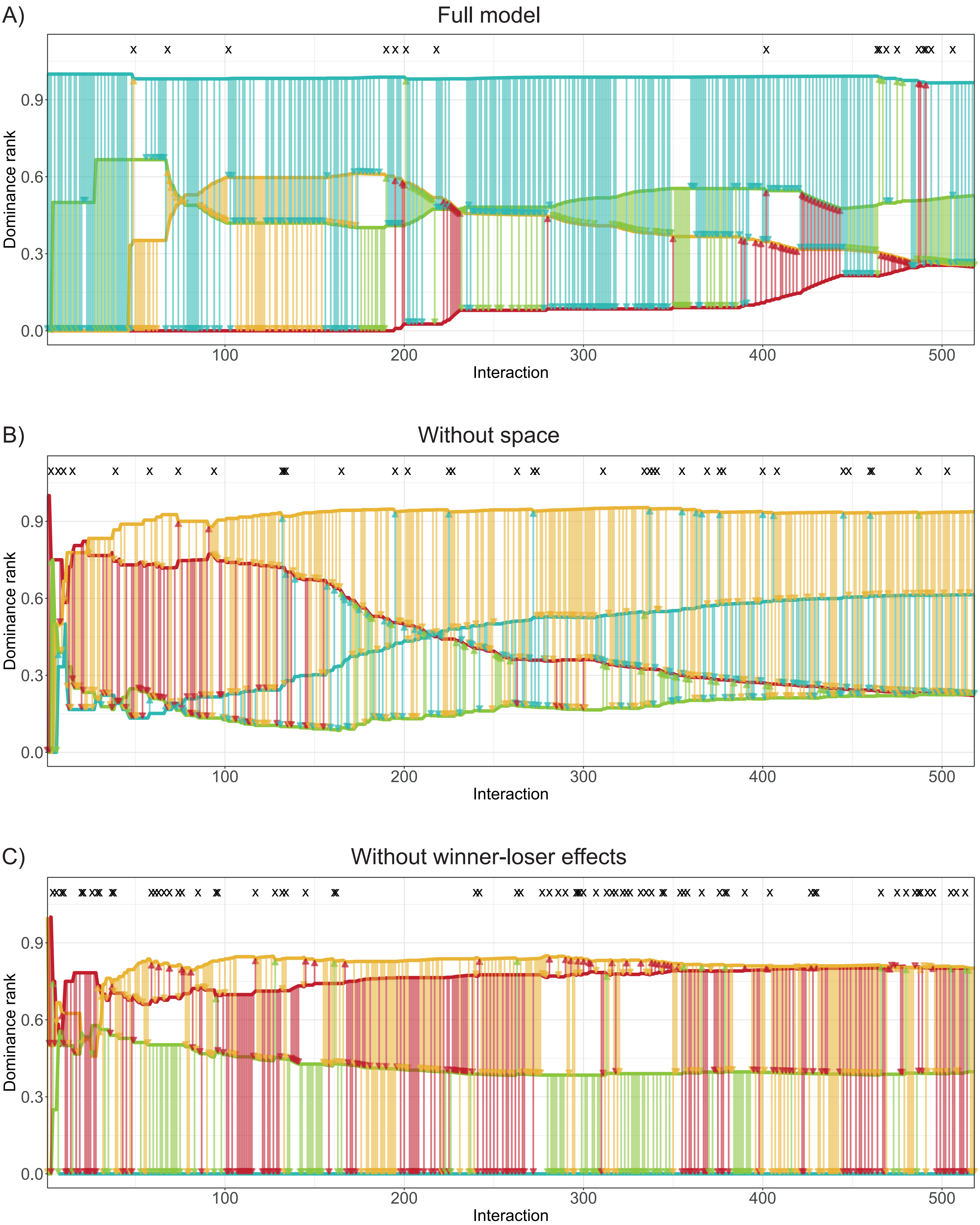

Appendix 2, Run 4. Music notation graph of rank development over interaction count for A) the full DomWorld model, B) without the spatial component and C) without the winner-loser effect. The horizontal lines represent the rank of each individual based on the average dominance index. The vertical arrows represent fights pointing from the winner to the loser, in the colour of the winner. Pair-flips are marked with an ‘X’ at the top of the graph. Rank changes are shown as crossing horizontal lines.

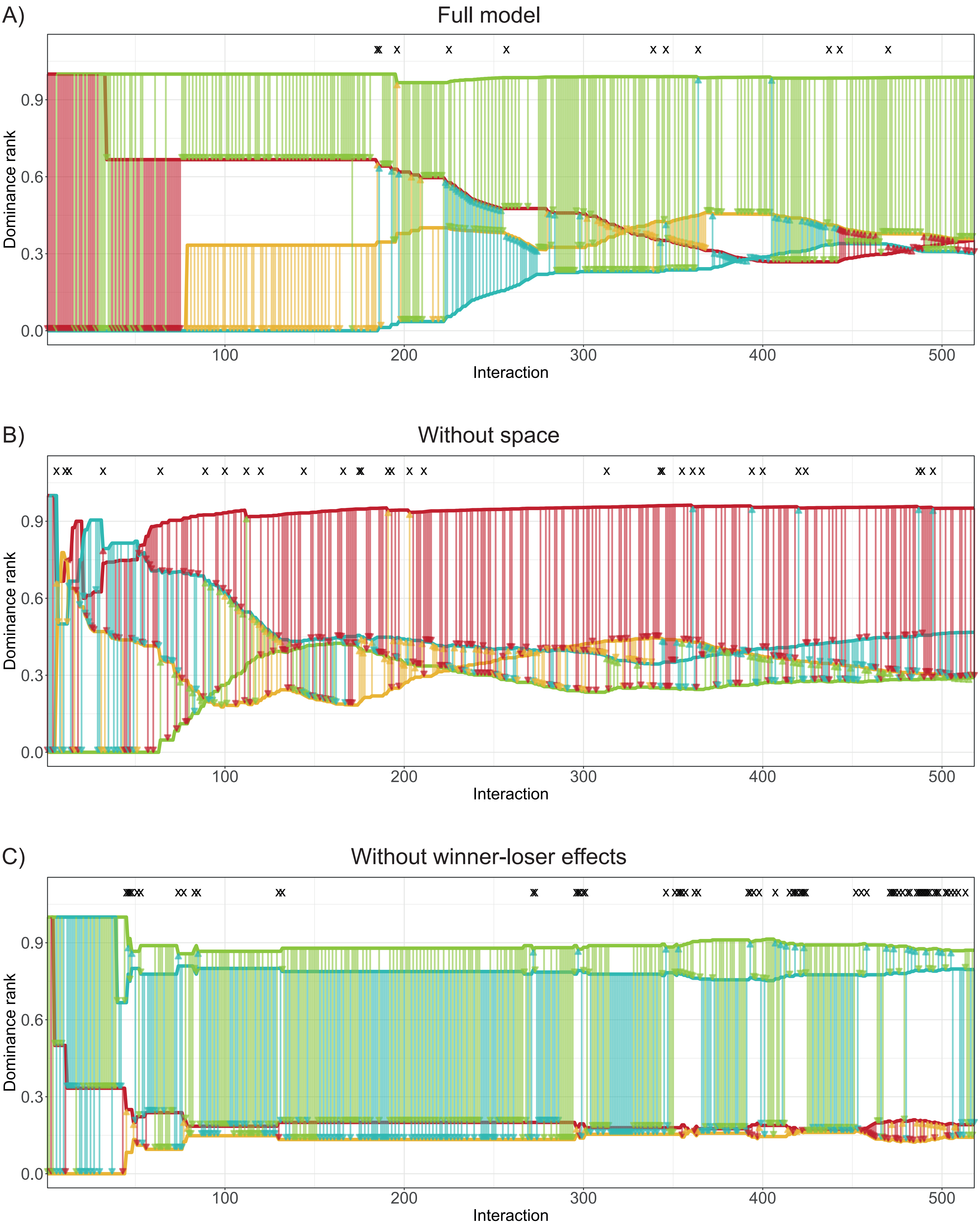

Appendix 2, Run 5. Music notation graph of rank development over interaction count for A) the full DomWorld model, B) without the spatial component and C) without the winner-loser effect. The horizontal lines represent the rank of each individual based on the average dominance index. The vertical arrows represent fights pointing from the winner to the loser, in the colour of the winner. Pair-flips are marked with an 'X' at the top of the graph. Rank changes are shown as crossing horizontal lines.

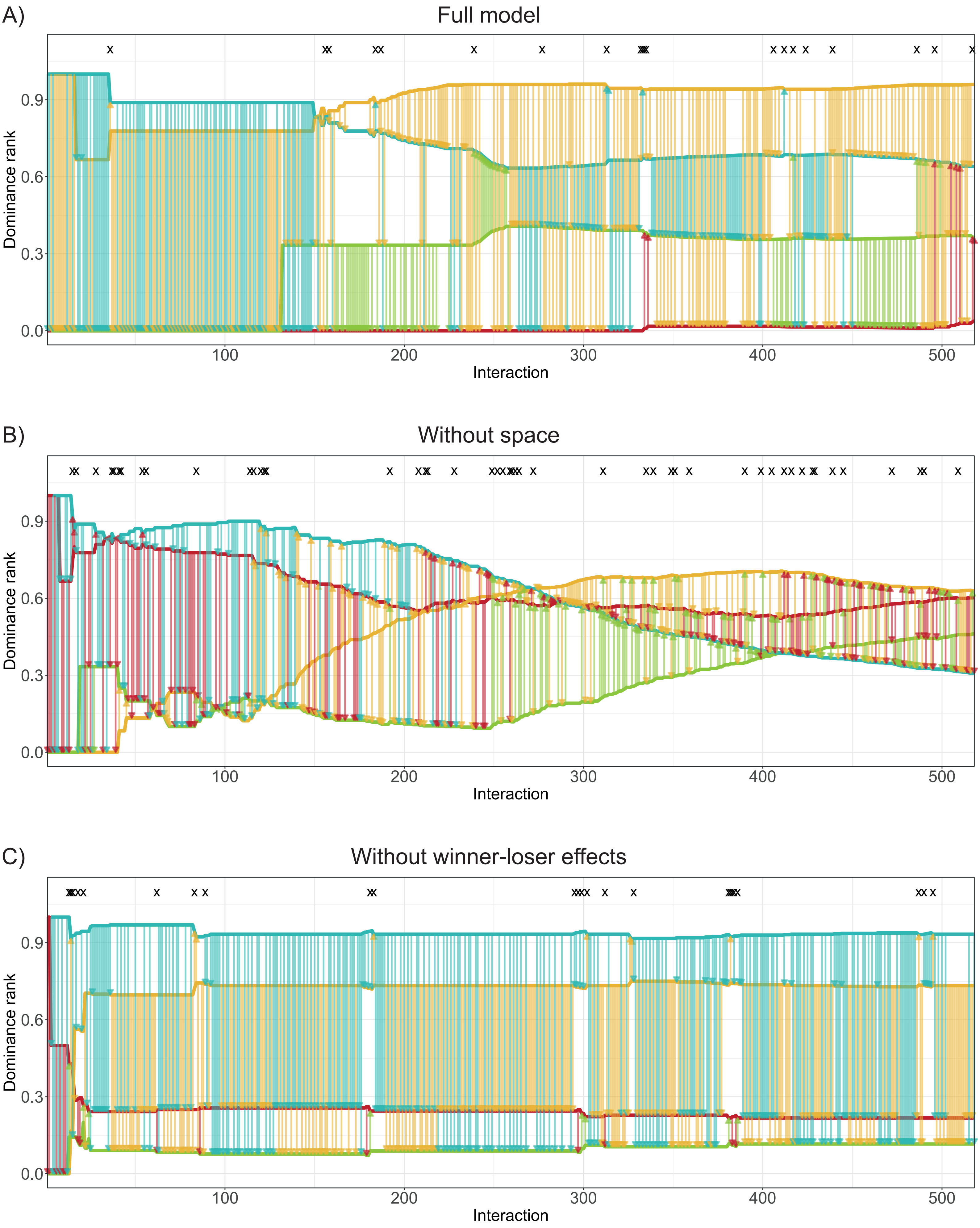

Appendix 2, Run 6. Music notation graph of rank development over interaction count for A) the full DomWorld model, B) without the spatial component and C) without the winner-loser effect. The horizontal lines represent the rank of each individual based on the average dominance index. The vertical arrows represent fights pointing from the winner to the loser, in the colour of the winner. Pair-flips are marked with an ‘X’ at the top of the graph. Rank changes are shown as crossing horizontal lines.

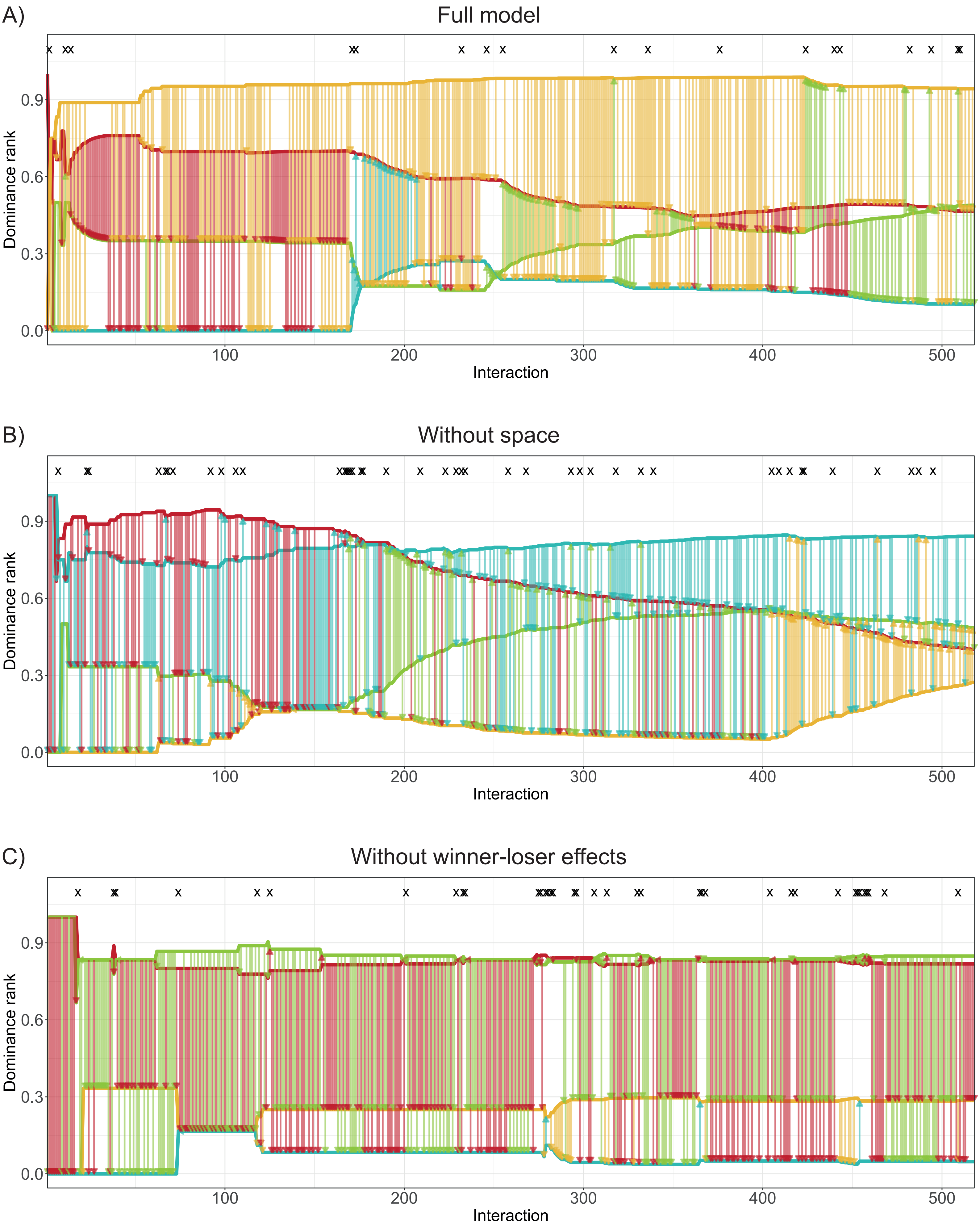

Appendix 2, Run 7. Music notation graph of rank development over interaction count for A) the full DomWorld model, B) without the spatial component and C) without the winner-loser effect. The horizontal lines represent the rank of each individual based on the average dominance index. The vertical arrows represent fights pointing from the winner to the loser, in the colour of the winner. Pair-flips are marked with an 'X' at the top of the graph. Rank changes are shown as crossing horizontal lines.

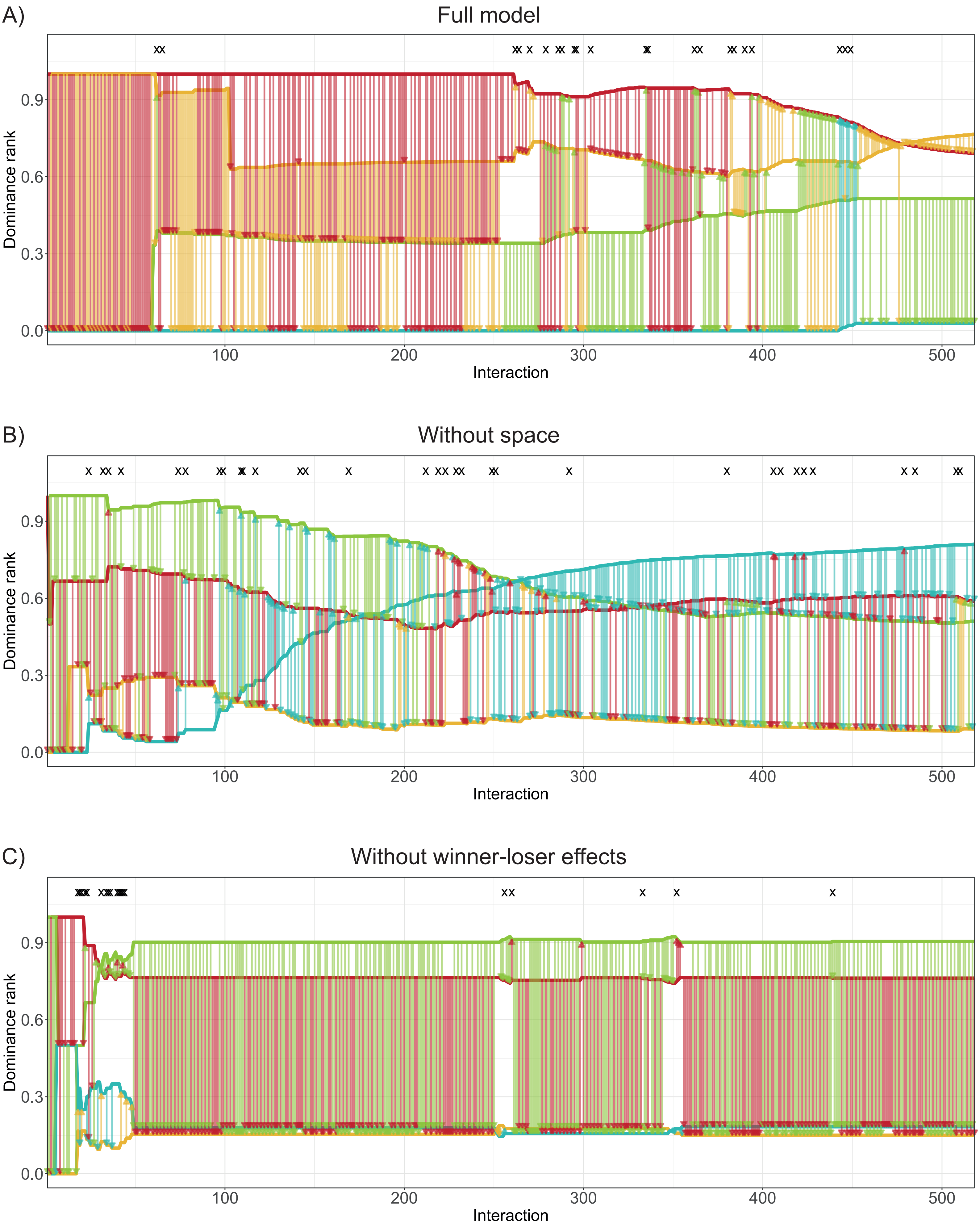

Appendix 2, Run 8. Music notation graph of rank development over interaction count for A) the full DomWorld model, B) without the spatial component and C) without the winner-loser effect. The horizontal lines represent the rank of each individual based on the average dominance index. The vertical arrows represent fights pointing from the winner to the loser, in the colour of the winner. Pair-flips are marked with an 'X' at the top of the graph. Rank changes are shown as crossing horizontal lines.

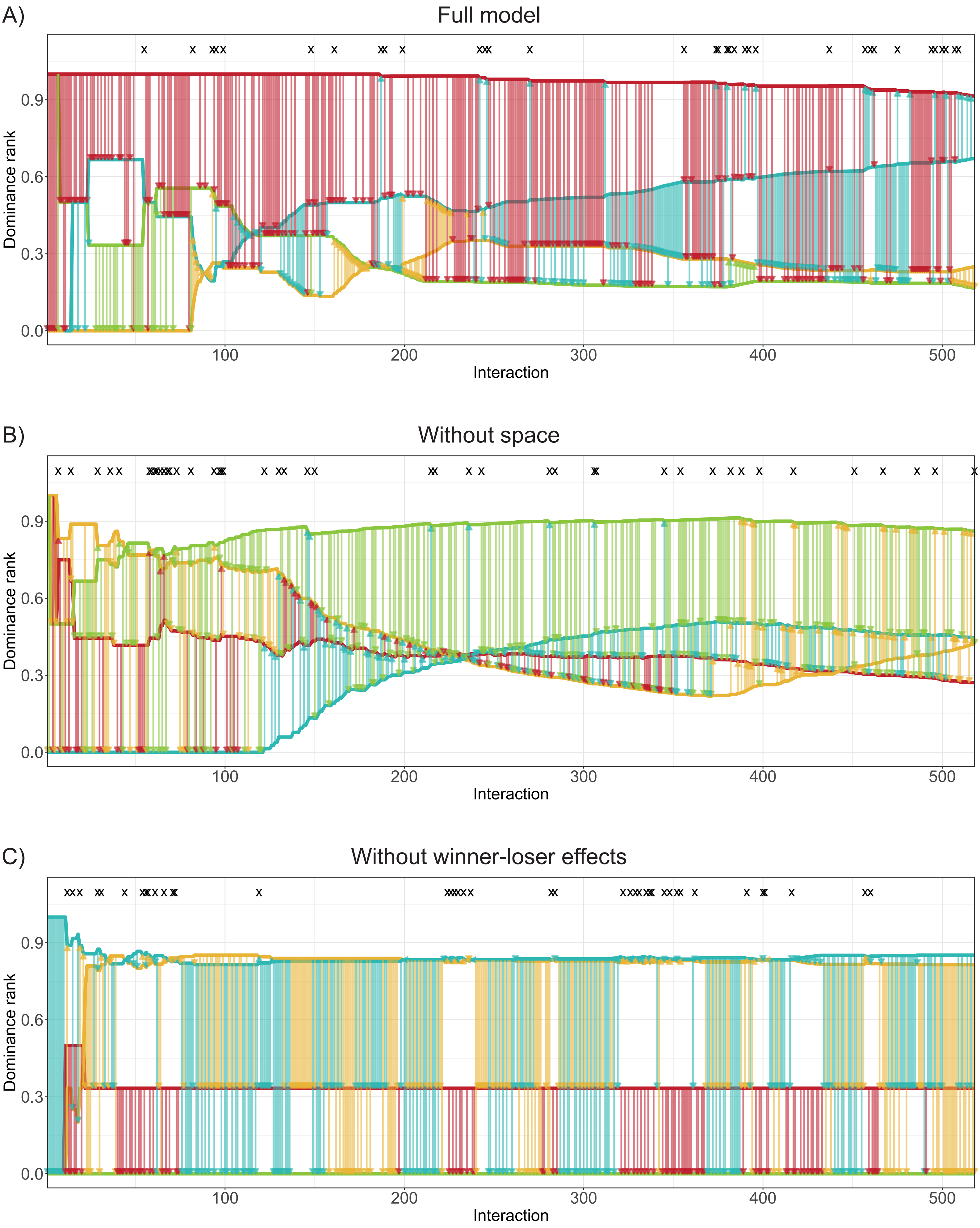

Appendix 2, Run 9. Music notation graph of rank development over interaction count for A) the full DomWorld model, B) without the spatial component and C) without the winner-loser effect. The horizontal lines represent the rank of each individual based on the average dominance index. The vertical arrows represent fights pointing from the winner to the loser, in the colour of the winner. Pair-flips are marked with an ‘X’ at the top of the graph. Rank changes are shown as crossing horizontal lines.

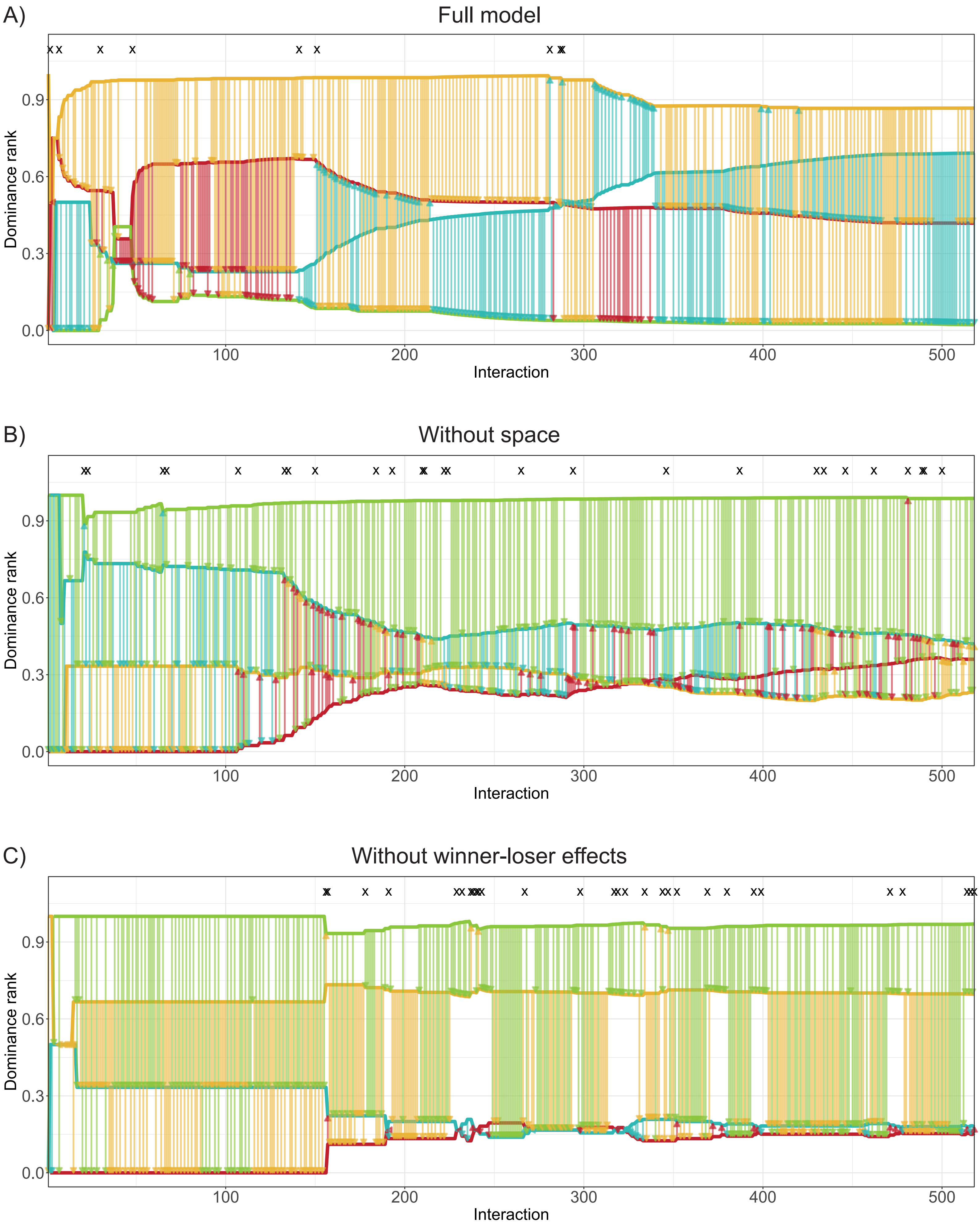

Appendix 2, Run 10. Music notation graph of rank development over interaction count for A) the full DomWorld model, B) without the spatial component and C) without the winner-loser effect. The horizontal lines represent the rank of each individual based on the average dominance index. The vertical arrows represent fights pointing from the winner to the loser, in the colour of the winner. Pair-flips are marked with an ‘X’ at the top of the graph. Rank changes are shown as crossing horizontal lines.

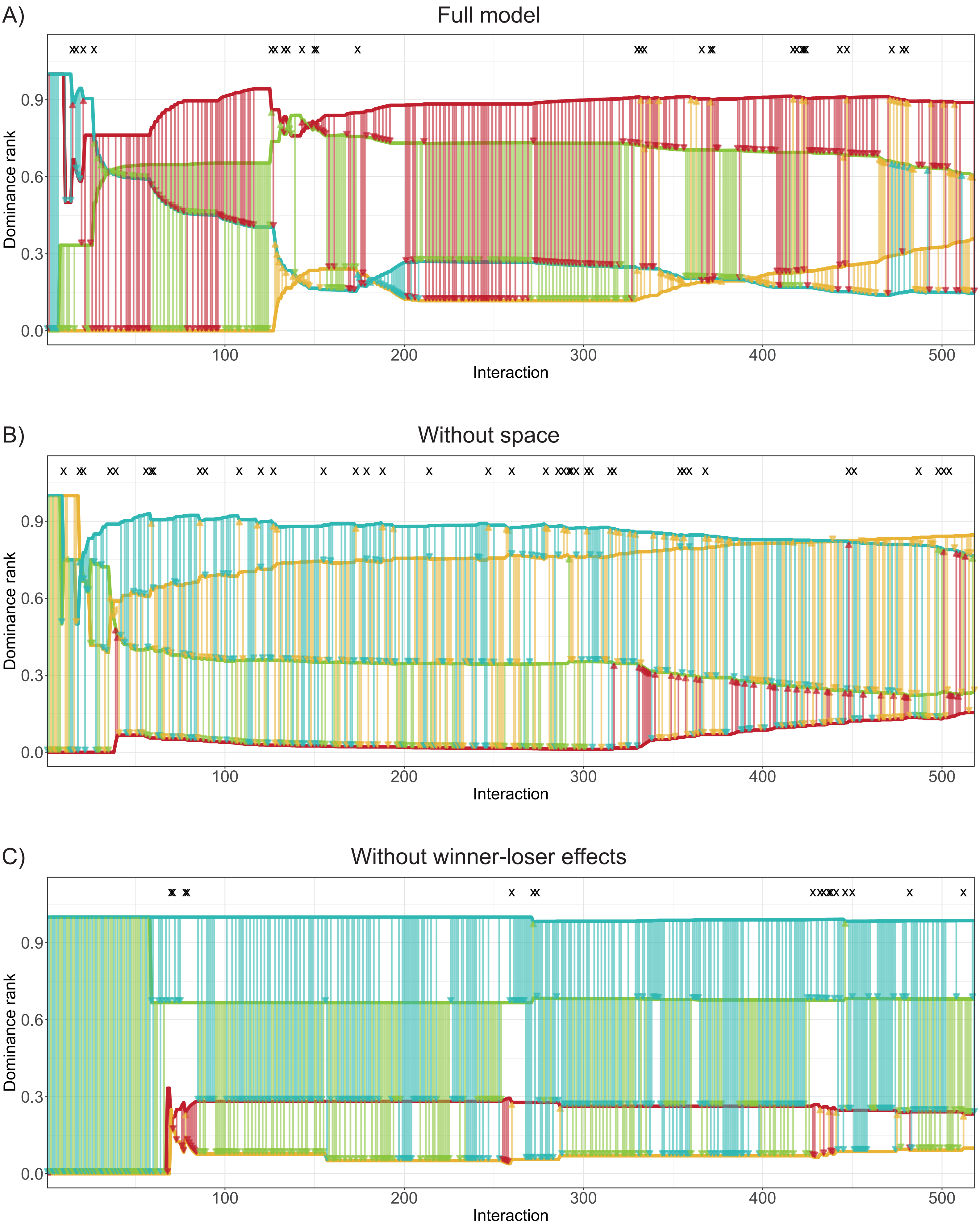

Appendix 2, Run 11. Music notation graph of rank development over interaction count for A) the full DomWorld model, B) without the spatial component and C) without the winner-loser effect. The horizontal lines represent the rank of each individual based on the average dominance index. The vertical arrows represent fights pointing from the winner to the loser, in the colour of the winner. Pair-flips are marked with an 'X' at the top of the graph. Rank changes are shown as crossing horizontal lines.

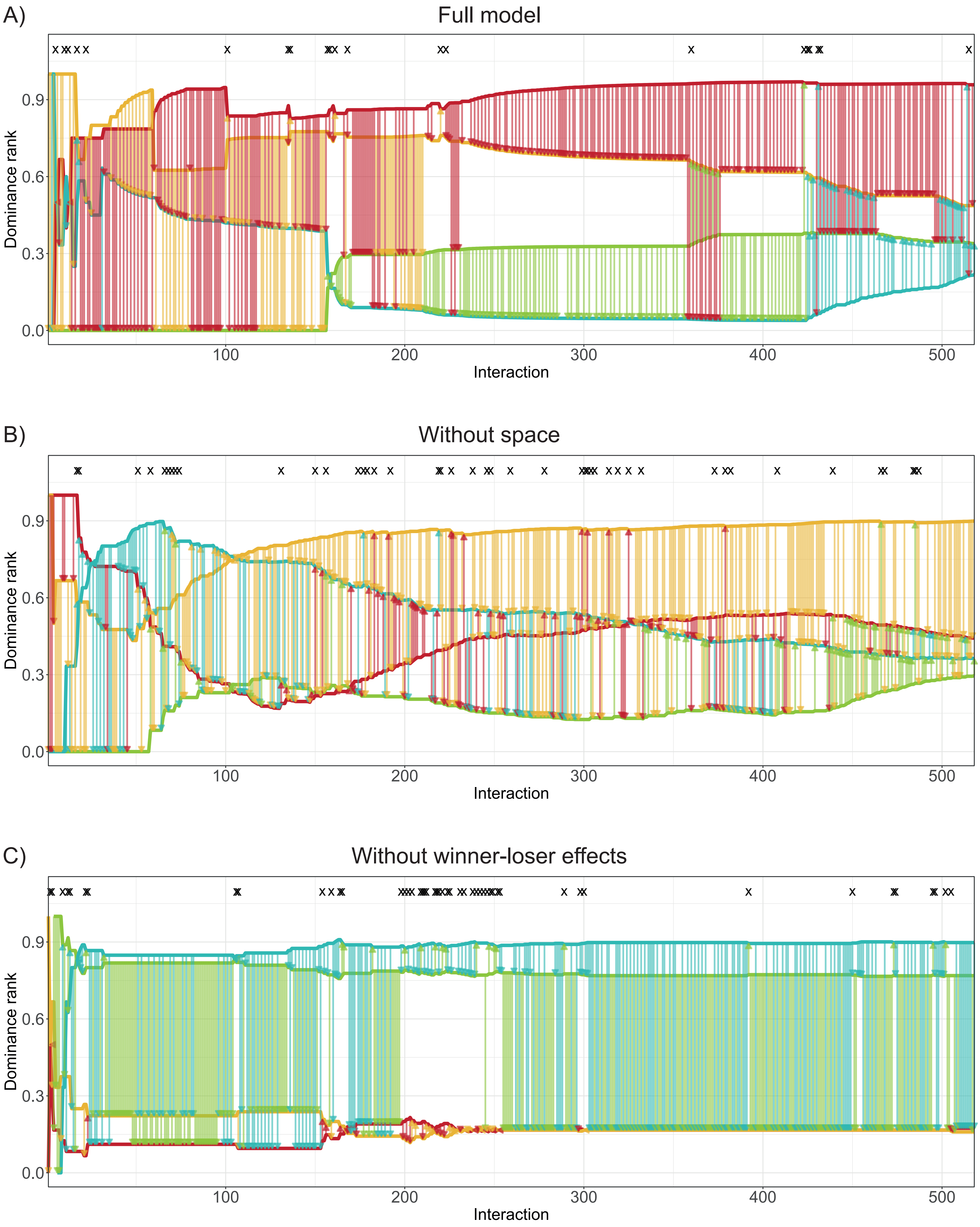

Appendix 2, Run 12. Music notation graph of rank development over interaction count for A) the full DomWorld model, B) without the spatial component and C) without the winner-loser effect. The horizontal lines represent the rank of each individual based on the average dominance index. The vertical arrows represent fights pointing from the winner to the loser, in the colour of the winner. Pair-flips are marked with an ‘X’ at the top of the graph. Rank changes are shown as crossing horizontal lines.

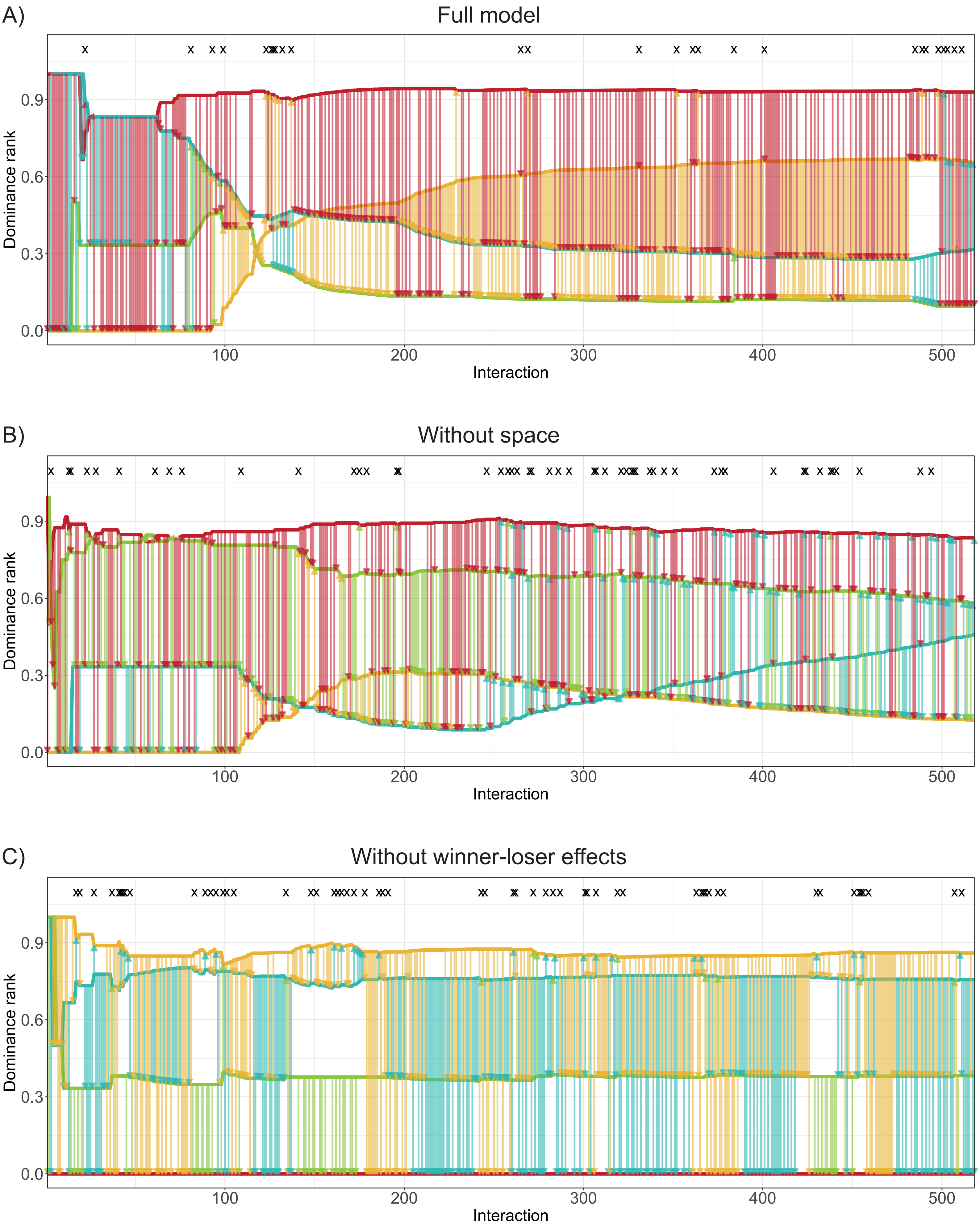

Appendix 2, Run 13. Music notation graph of rank development over interaction count for A) the full DomWorld model, B) without the spatial component and C) without the winner-loser effect. The horizontal lines represent the rank of each individual based on the average dominance index. The vertical arrows represent fights pointing from the winner to the loser, in the colour of the winner. Pair-flips are marked with an ‘X’ at the top of the graph. Rank changes are shown as crossing horizontal lines.

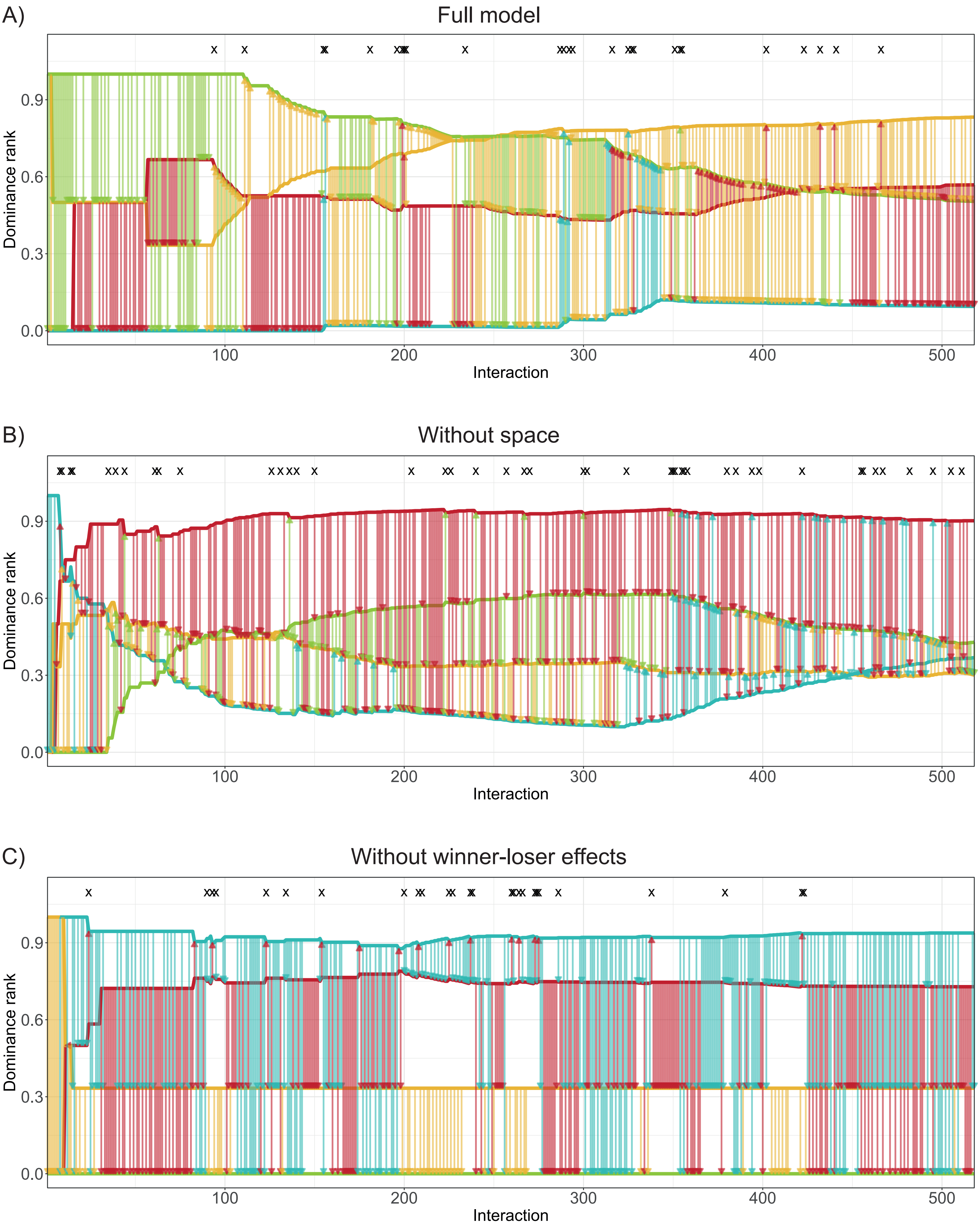

Appendix 2, Run 14. Music notation graph of rank development over interaction count for A) the full DomWorld model, B) without the spatial component and C) without the winner-loser effect. The horizontal lines represent the rank of each individual based on the average dominance index. The vertical arrows represent fights pointing from the winner to the loser, in the colour of the winner. Pair-flips are marked with an 'X' at the top of the graph. Rank changes are shown as crossing horizontal lines.
